# Haemodynamic and biomechanical biomarker analysis of carotid fibromuscular dysplasia via fluid–structure interaction

**DOI:** 10.1007/s10237-026-02099-x

**Published:** 2026-07-21

**Authors:** Kaveh Moghadasi, Mergen H. Ghayesh, Eric Hu, Shahid Hussain, Marco Amabili, Robert Fitridge, Jiawen Li

**Affiliations:** 1 School of Electrical and Mechanical Engineering, Adelaide University, Adelaide, SA 5005 Australia; 2 School of Information Technology and Systems, University of Canberra, Canberra, ACT Australia; 3 School of Engineering, Westlake University, Hangzhou, China; 4 Department of Mechanical Engineering, McGill University, Montreal, H3A 0C3 Canada; 5 Vascular and Endovascular Service, Royal Adelaide Hospital, Adelaide, Australia; 6 Discipline of Surgery, Adelaide University, Adelaide, Australia; 7 Basil Hetzel Institute for Translational Health Research, The Queen Elizabeth Hospital, Adelaide, Australia; 8 Institute for Photonics and Advanced Sensing, Adelaide University, Adelaide, SA 5005 Australia

**Keywords:** Carotid artery, Fibromuscular dysplasia disease, Fluid–structure interaction, Anisotropic artery tissue, Wall shear biomarkers

## Abstract

This study aims to investigate the biomechanical behaviour of the carotid artery in patients with fibromuscular dysplasia (FMD) disease. Carotid FMD is an arterial disease lacking either inflammatory or atherosclerotic pathology, which is characterised by segmental disruptions in arterial wall architecture. It is of considerable interest to examine carotid FMD haemodynamics for the identification of clinically meaningful biomechanical biomarkers. Thus, a two-way coupled three-dimensional (3D) fluid–structure interaction (FSI) model was developed that integrates patient-specific vascular geometries, non-Newtonian turbulent blood flow, an orthotropic hyperelastic representation of the arterial wall, and a Windkessel boundary formulation, with emphasis on characterising haemodynamic biomarkers and biomechanical wall responses. The results showed distinct severity-dependent trends among healthy, focal, non-focal, and severe non-focal carotid geometry types. FMD cases exhibited increased velocities and wall shear stresses, while their pressure gradients at the distal end decreased. Additionally, elevation of OSI (oscillatory shear index) and RRT (relative residence time) values was observed in each FMD model indicating higher levels of flow disruption, oscillatory shear, and localised flow stagnation. Non-focal phenotypes showed the largest radial deformation, whereas the focal configuration displayed the highest von-Mises stresses. These findings indicate that as FMD progresses through increasing complexity in its morphological structure, it will be subjected to increasingly adverse haemodynamics and mechanical forces, which are likely to promote endothelial dysfunction and further progression of the disease.

## Introduction

**Fig. 1:**
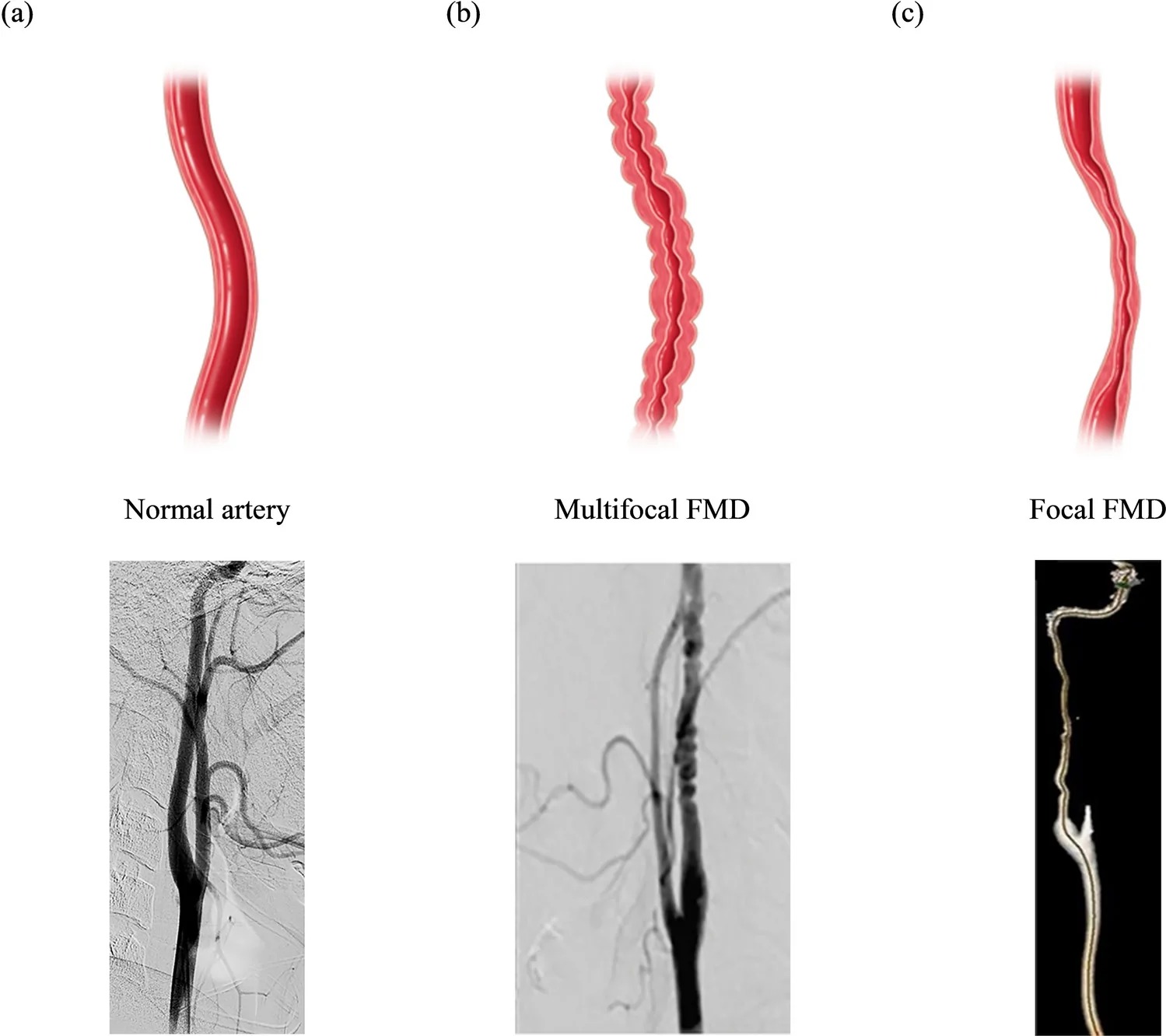
Different types of FMD compared to healthy artery in cerebrovascular system. **a** Normal carotid artery (Wang et al. 2022); **b** multifocal FMD in carotid artery (Green et al. 2020); **c** focal FMD in carotid artery (Mazza et al. 2009). Reprinted with permission, under the Creative Commons

Fibromuscular dysplasia (FMD) is a relatively common non-inflammatory and non-atherosclerotic arterial disease (affecting 3–5% of adult women) that can cause stenosis, aneurysm formation, dissection, and tortuosity, most commonly in the carotid and renal arteries (d’Escamard et al. 2024; Slovut and Olin 2004). This disease primarily affects women from age 20–60, but may also occur in infants and children, men, and the elderly (Pascual et al. 2005). In the cerebrovascular system, FMD is increasingly recognised as a cause of transient ischaemic attacks (TIAs) and stroke in young and middle-aged individuals without classical cardiovascular risk factors (Mazza et al. 2009). Extracranial FMD predominantly occurs in the internal carotid artery at the level of the C1 and C2 vertebrae (considerably higher than the carotid artery bifurcation), with historical reports indicating carotid artery involvement in approximately 25–30% of FMD patients. Figure 1 depicts the two predominant morphological patterns of carotid FMD, characterised by segmental arterial narrowing and focal dilatation, in comparison with a healthy carotid artery. These deformities can impair blood flow and may contribute to significant health risks. Diagnostic imaging identifies two distinct forms of fibromuscular dysplasia (Narula et al. 2018; Plouin et al. 2007): (i) multifocal FMD (shown in Fig. 1b), the most common subtype, is characterised by repeated cycles of arterial constriction and bulging, producing the “string-of-beads” appearance, (ii) focal FMD (shown in Fig. 1c), which is less common, presents a single, localised area of arterial narrowing or dilatation.

FMD can be detected using duplex ultrasound, computed tomography (CT) angiography, or magnetic resonance angiography (MRA) (Olin and Sealove 2011). Because FMD predominantly affects more distal arterial segments than those typically involved in atherosclerotic plaque formation, it is often not visualised by ultrasound and frequently remains underdiagnosed. However, when FMD is seen on ultrasound, elevated arterial flow velocities are often demonstrated. FMD is also associated with a broad spectrum of clinically significant complications. Among the most severe is carotid artery dissection, which may progress to arterial rupture. Dissection may result in devastating outcomes for the typically young and otherwise healthy FMD patient (Dziewas et al. 2003). FMD can compromise the arterial lumen, resulting in reduced cerebral perfusion and increasing the risk of thrombus formation with subsequent distal embolisation (Kesav et al. 2023). Moreover, it may coexist with other vascular abnormalities that further complicate diagnosis and clinical management (Touzé et al. 2019). These include atherosclerotic occlusion of the carotid bifurcation, as well as extracranial carotid artery aneurysms and concurrent involvement of the vertebral arteries (LÜSCHER et al. 1987).

Cerebrovascular FMD is strongly linked to cervical (carotid/vertebral) artery dissections and TIA/stroke in young to middle-aged adults where current risk tools are weakest (Green et al. 2020). Contemporary vascular imaging modalities, including Doppler ultrasonography, computed tomography angiography (CTA), magnetic resonance angiography (MRA), and catheter-based angiography, play a crucial role in the diagnosis of carotid artery pathologies. Beyond FMD, these techniques are routinely employed in the evaluation of other carotid diseases, such as atherosclerotic carotid stenosis (Brinjikji et al. 2016; Saxena et al. 2019), carotid web (Kim et al. 2019; Madaelil et al. 2019), and carotid aneurysms (Cornwall et al. 2021; Rosset et al. 2000), providing valuable anatomical and morphological information. While imaging enables visualisation of luminal narrowing, arterial tortuosity, and vessel wall irregularities, it remains inherently limited in its ability to quantitatively characterise the underlying haemodynamic significance of these lesions. In contrast, quantitative haemodynamic assessment methods offer the capability to evaluate flow disturbances, pressure gradients, and wall-induced stresses that are not directly observable from imaging diagnosis. Such haemodynamic markers have been shown to play a critical role in determining lesion severity and clinical risk across diverse carotid pathologies, highlighting the need for modelling strategies that move beyond purely geometric descriptors towards a mechanistic understanding of blood flow-artery interactions.

Computational fluid dynamics (CFD) modelling has therefore emerged as a powerful tool for quantifying flow-related biomarkers in patient-specific carotid geometries. However, despite the recognised involvement of the carotid arteries in FMD, its computational investigations remain exceedingly scarce. To date, the only dedicated patient-specific modelling study has focused on FMD-related renal artery stenosis, demonstrating the feasibility of an image-based CFD framework to non-invasively quantify trans-stenotic pressure drops and fractional flow reserve as clinically meaningful indicators of lesion severity (Soliveri et al. 2025). While this work provides an important proof of concept for the application of computational haemodynamics in FMD, its exclusive focus on the renal vasculature limits direct translation to the carotid circulation, where arterial geometry, wall mechanics, flow pulsatility, and cerebrovascular risk profiles are fundamentally different. Consequently, the haemodynamic mechanisms underlying carotid FMD, and their interaction with arterial wall deformation, remain largely unexplored, highlighting a critical gap in the current literature.

In this context, quantitative haemodynamic analysis allows for an integrated approach to investigate a broad spectrum of carotid artery diseases characterised by abnormal flow conditions. The use of flow-derived biomechanical markers in assessing local haemodynamic environments across diverse pathologies, including atherosclerotic carotid stenosis, carotid web, and carotid aneurysms, has provided valuable insight into the mechanisms involved in initiation, progression, and thromboembolic risk in anatomically distinct carotid lesions. Furthermore, the ability to assess the interaction of blood flow patterns and the arterial wall using these biomarkers enables a comparative quantitative basis for evaluating different types of carotid disease within a unified modelling paradigm (Compagne et al. 2019; Gharahi et al. 2016; Moghadasi et al. 2024a, 2024b; Sousa et al. 2016). For example, numerous studies have demonstrated that fluctuations in blood flow velocity and wall shear stress (WSS) are crucial factors in regulating both the onset and progression of atherosclerotic plaque formation within the carotid artery (Conti et al. 2016; Moerman et al. 2022; Zhou et al. 2023b). Regions of low WSS are associated with plaque initiation and growth, whereas elevated WSS has been implicated in triggering plaque rupture (Gharahi et al. 2016; Moghadasi et al. 2024a).

FSI modelling provides an overall framework for capturing the influence of arterial wall mechanics on blood flow dynamics. By explicitly accounting for vessel deformability and the two-way coupling between pulsatile flow and arterial wall motion, FSI represents a more physiologically accurate modelling of local haemodynamic behaviour in complex vascular geometries. This integrative approach facilitates the identification of haemodynamically vulnerable regions, which could lead to the early diagnosis of conditions that may contribute to a high risk of stroke, and therefore, precision-targeted treatment to reduce this risk.

Zouggari (Zouggari et al. 2018) developed computational models of healthy and stenotic carotid arteries to explore the effects of plaque formation on haemodynamic forces. The application of FSI revealed that plaque presence significantly lowers WSS downstream and promotes localised flow recirculation, with FSI offering enhanced insight into wall deformation dynamics. In a study conducted by Lopes (Lopes et al. 2019), rigid-wall CFD and elastic wall FSI models of carotid blood flow were compared and it was demonstrated that FSI more accurately predicts velocity and wall shear stress by accounting for vessel elasticity. It was found that the carotid sinus is the region most susceptible to atherosclerosis, due to low values of time-averaged wall shear stress. These low shear stress conditions, especially when visualised using FSI models that account for arterial wall elasticity, correspond to areas of flow recirculation and disturbance, which are known to promote endothelial dysfunction and plaque development. These findings are consistent with clinical carotid studies showing that regions exposed to low and oscillatory wall shear stress are associated with plaque localisation, increased intima-media thickness, and longitudinal plaque progression, particularly at the carotid bifurcation (Carallo et al. 2016; Irace et al. 2004; Ku et al. 1985; Strecker et al. 2021).

Several studies have directly compared Newtonian and non-Newtonian formulations in carotid artery simulation. Boyd and Buick (2007) demonstrated that blood rheology can significantly influence near-wall shear stress predictions in carotid models, which are critical for assessing atherosclerosis-related haemodynamics. More recent investigations further support these findings. Morbiducci showed that the influence of non-Newtonian rheology is most pronounced in low shear and disturbed-flow regions within the carotid bifurcation. Lopes et al. (2020), in an FSI framework, reported that Carreau-type non-Newtonian models alter haemodynamic quantities such as wall shear stress (WSS), particularly in complex flow environments. A previous comprehensive review (Moghadasi et al. 2026) indicates that non-Newtonian models provide improved fidelity in predicting haemodynamic indices such as time-averaged wall shear stress (TAWSS), oscillatory shear index (OSI), and relative residence time (RRT), especially in stenotic and post-stenotic carotid flows. Likewise, Liu et al. (2025) performed a systematic comparison of viscosity models in carotid artery stenosis and reported statistically significant differences in key haemodynamic indices, showing that Newtonian model tends to overestimate TAWSS, OSI, and RRT. Bantwal et al. (2021) also modelled a plaque within the carotid sinus to simulate luminal stenosis and examined resulting haemodynamic parameters such as oscillatory shear index (OSI) and relative residence time (RRT), both indicative of disturbed blood flow. Compared to the healthy arterial model, the stenotic models showed elevated OSI and RRT values, which suggest an increased risk of thrombus formation in the event of plaque rupture. Despite extensive application of CFD and FSI methods in atherosclerotic carotid artery disease, carotid webs, and carotid aneurysms, haemodynamic and FSI modelling of carotid FMD remains unexplored. Current modelling approaches rarely incorporate the dynamic interaction between blood flow and vessel wall deformation in FMD, especially in the carotid artery.

Cerebrovascular FMD has a range of manifestations, from being completely asymptomatic to having a wide variety of nonspecific symptoms such as headache, dizziness, vertigo, and syncope (Kesav et al. 2023; Touzé et al. 2010). Although in some cases, more specific neurological symptoms such as TIA, ischaemic stroke, Horner’s syndrome, and cranial nerve palsies constitute the initial clinical presentation, especially when the carotid or vertebral artery is involved. These symptoms generally result from haemodynamically significant stenoses or occlusions, causing abnormal flow patterns and wall shear stress (WSS) distributions, which may lead to endothelial dysfunction and localised plaque formation.

Given the inherent limitations of current imaging techniques or rigid-wall CFD frameworks to capture these biomechanical disturbances, the necessity for a fully coupled FSI approach becomes evident (Gornik et al. 2019). There is currently no established non-invasive method to quantitatively assess the haemodynamic significance of carotid FMD, particularly with respect to flow disturbance, translesional pressure gradients, and wall shear stress abnormalities implicated in cerebrovascular risk. Therefore, this study aims, for the first time, to evaluate the feasibility of an image-based fluid–structure interaction (FSI) framework to non-invasively characterise the haemodynamic impact of carotid FMD and identify flow-derived biomarkers associated with disease severity and stroke risk (Persu et al. 2012). We have developed patient-specific FSI models that allow the routine use of CTA/MRA images to create actionable risk maps and to predict areas of potential dissection and thromboembolism. We also utilise the models to provide a stratification scheme for monitoring patients who have been identified to be at high-risk, and finally, our models enable us to virtually test different angioplasty approaches prior to performing actual treatment.

## Materials and methods

**Fig. 2:**
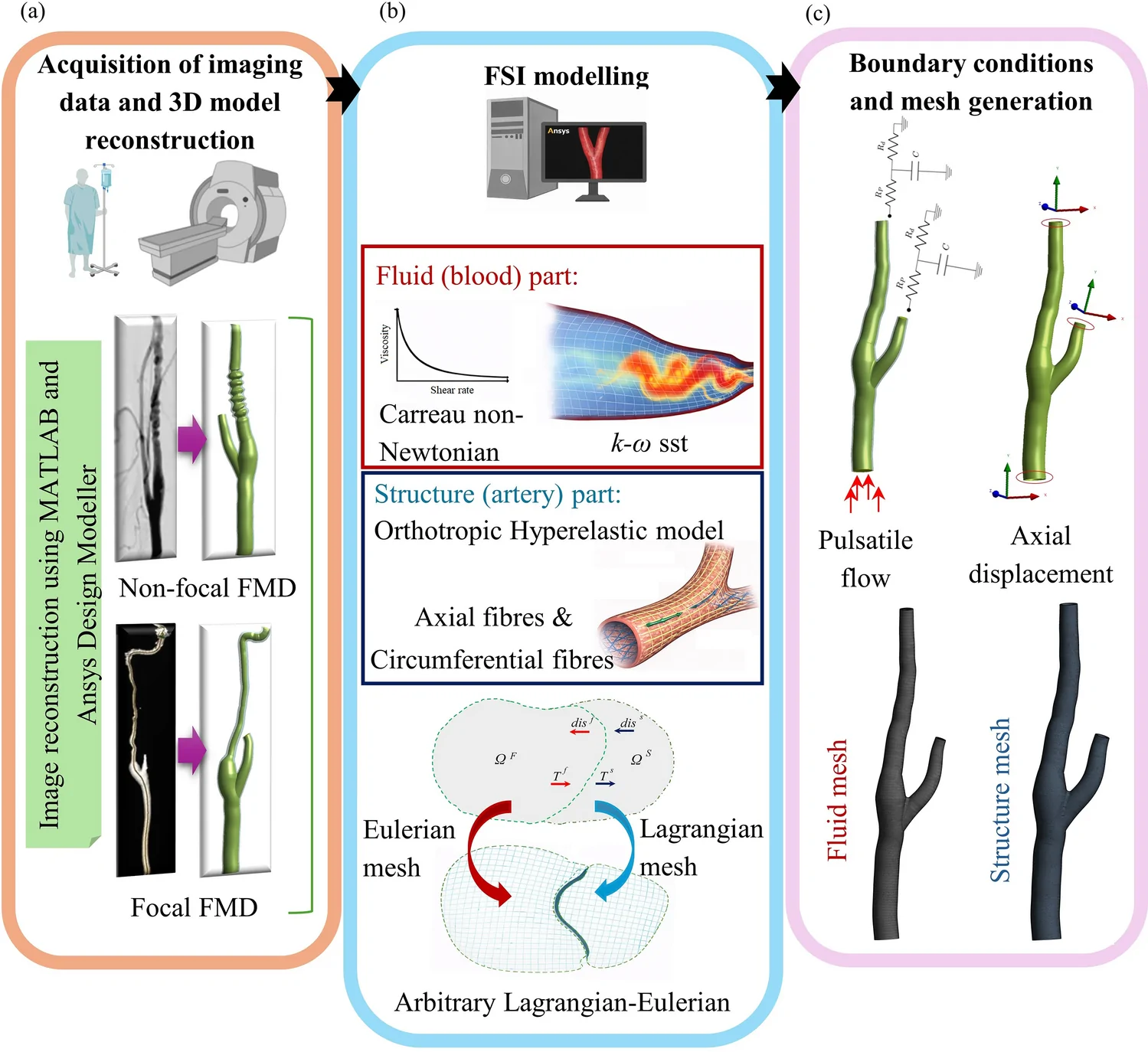
Schematic illustration of the FSI modelling framework for the carotid artery FMD; **a** patient-specific geometry reconstruction based on imaging data reported in the literature; **b** definition of material models for the FSI simulations including the fluid (blood) and solid (arterial wall) domains, together with arbitrary Lagrangian–Eulerian (ALE) formulation; **c** specifications of boundary conditions and mesh characteristics for the developed computational models

This section outlines the sequential stages of FSI modelling applied to FMD in the carotid artery. The complete workflow, ranging from patient-specific geometry reconstruction based on CTA data to the FSI implementation, including the definition of fluid (blood) and solid (arterial wall) domains, the arbitrary Lagrangian–Eulerian (ALE) formulation, boundary conditions, and mesh characteristics, is summarised in Fig. 2. Accordingly, the subsequent subsections describe each modelling component in detail to ensure methodological clarity.

### Geometry

This study employs patient-derived CTA data from carotid arteries affected by FMD to reconstruct three-dimensional geometries for detailed computational analysis. Figure 2a presents two reconstructed geometries representing non-focal and focal FMD. The fluid (blood) domain was initially extracted using MATLAB 2021R2, after which a computer-aided design (CAD) workflow in ANSYS workbench 2023R2 was employed to construct the three-dimensional volumetric blood domain through surface generation and Boolean operations. Subsequently, the solid (arterial wall) domain was constructed by offsetting the fluid geometry with a uniform wall thickness of 0.8 mm in non-diseased regions. It is important to note that, in FMD-affected segments, histopathological evidence indicates degeneration of medial smooth muscle cells and their replacement by fibrotic tissue rather than classical wall hypertrophy (Saba et al. 2010; Varennes et al. 2015). Accordingly, the diseased regions were represented using an effective wall thickness to account for structural remodelling and reduced arterial compliance, consistent with imaging-based modelling assumptions. To examine the haemodynamic alterations associated with disease severity, an additional non-focal FMD model representing a more advanced case was also reconstructed. All haemodynamic results were benchmarked against a healthy carotid artery model. The models considered in this study are denoted by the following abbreviations: (i) FMD-F (focal carotid artery FMD), (ii) FMD-NF (non-focal carotid artery FMD), (iii) FMD-SNF (severe case of non-focal carotid artery FMD).

### Governing equations

In this study, the haemodynamic behaviour of blood flow is modelled based on the Navier–Stokes equations governing incompressible fluid motion (Cabaniss et al. 2025). Within the FSI framework (shown in Fig. 2b), the fluid domain is resolved using a modified form of the momentum equation incorporating the moving mesh velocity formulation, in conjunction with the continuity equation, as presented in Eq. 1 (Temam 2024; Wu et al. 2022):

1 $$\frac{d}{dt}\int_{\Omega } {\rho v_{i} d\Omega } + \int_{S} {\rho v_{i} (v_{j} - v_{bj} } )n_{j} dS = \int_{S} {(\tau_{ij} - p\delta_{ij} } )n_{j} dS + \int_{\Omega } {\rho b_{i} \partial \Omega } ,$$

where *ρ* is the fluid density, *v*_*i*_ is the *i*-th velocity component, *τ*_*ij*_ denotes the viscous stress tensor, *δ*_*ij*_ represents Kronecker delta, *p* is pressure, *b*_*i*_ is the body force per unit volume (e.g. gravity), *v*_*j*_ is the fluid velocity vector, and *v*_*bj*_ represents the velocity of the moving boundary, *Ω* is the fluid domain, and *S* represents the moving boundary.

The arterial wall is modelled as a deformable solid governed by the balance of linear momentum in a Lagrangian description (Belytschko 1980; Hou et al. 2012):

2 $$\rho _{s} \frac{{\partial ^{2} {\mathbf{u}}_{s} }}{{\partial t^{2} }} = \nabla .\sigma _{s} + \rho _{s} {\mathbf{f}}_{s} ,$$

where *ρ*_s_, **u**_s_, *σ*_s,_ and **f**_s_ denote the arterial wall density, displacement vector, Cauchy stress tensor, and body force, respectively. To address the limitations of purely Lagrangian and Eulerian formulations in FSI problems, the ALE method is employed, allowing the fluid mesh to deform consistently with the structural motion at the interface (Fandaros et al. 2025; Yan et al. 2025; Zare Jouneghani et al. 2025). Conservation of mass and linear momentum at the fluid–structure interface is enforced through kinematic and dynamic compatibility conditions (Paidoussis 1998; Wick 2013):

3 $${\mathbf{d}}_{{s,\Gamma }} = {\mathbf{d}}_{{f,\Gamma }} ,\quad {\mathbf{T}}_{{s,\Gamma }} = {\mathbf{T}}_{{f,\Gamma }} ,$$

ensuring displacement continuity (**d**) and traction equilibrium (**T**) between the fluid and solid domains. A partitioned coupling strategy is adopted, whereby interface displacements are transferred from the structural solver to the fluid solver, and the resulting fluid pressures and shear stresses are applied as nodal loads on the arterial wall.

### Blood properties

Blood was modelled as an incompressible, non-Newtonian fluid exhibiting pulsatile flow and turbulent characteristics. To accurately capture its physiological shear thinning behaviour, the Carreau viscosity model was employed (Cho and Kensey 1991):

4 $$\mu = \mu _{\infty } \left( {1 - \left[ {1 + \left( {\lambda \dot{\gamma }} \right)^{2} } \right]^{{\frac{{n - 1}}{2}}} } \right) + \mu _{0} \left[ {1 + \left( {\lambda \dot{\gamma }} \right)^{2} } \right]^{{\frac{{n - 1}}{2}}} .$$

Here, $\mu _{0}$ denotes the initial viscosity of the blood, specified as 0.056 Pa.s, while $\mu _{\infty }$ represents the infinite-shear viscosity, set at 0.00345 Pa.s. The parameter *λ* corresponds to the relaxation time (*λ* = 3.313), $\dot{\gamma }$ denotes the strain shear rate, and *n* is the dimensionless power index (*n* = 0.3568). The Carreau model is widely employed in arterial CFD modelling due to capturing the smooth change of blood viscosity with increasing shear rate, from high viscosity at low shear to near-constant viscosity at high shear. It ensures stable and realistic predictions across the wide shear-rate range encountered in large arteries (Akbar and Nadeem 2014). For turbulent properties, *k-ω SST* model was utilised due to combining the advantages of the *k-ω* model near the wall with the *k-ε* model outside the boundary layer. For a comprehensive discussion of various non-Newtonian models and turbulence characteristics, the reader is referred to a recently published review paper (Moghadasi et al. 2026).

### Haemodynamic indices (WSS, transWSS, OSI, and RRT)

WSS is a vector that represents the tangential force applied to the endothelial layer by the flowing blood. The magnitude and orientation of the WSS vector (*τ*_w_(*t*)) vary at every instant over the cardiac cycle for the pulsatile arterial flow. Thus, although TAWSS provides a means of quantifying the magnitude of shear exposure throughout the pulse cycle, it does not provide information about how often and to what degree the near-wall flow is subjected to directional changes. To take into consideration the multi-directional characteristics of wall shear behaviour, a complementary metric of WSS, called transverse wall shear stress (transWSS), has been introduced to specifically quantify the component of WSS acting perpendicular to the predominant flow direction. The cycle-averaged WSS vector can be expressed as follows (Hossain et al. 2024; Katritsis et al. 2007):

5 $$\overline{\tau } = \frac{1}{T}\int_{0}^{T} {\tau_{w} \left( t \right)} \;dt.$$

The unit direction vector is given by:

6 $$\hat{m} = \frac{{\overline{\tau }}}{{\left\| {\overline{\tau }} \right\|}}.$$

The instantaneous transverse component of WSS is then obtained by removing the projection of *τ*_*w*_ (*t*) onto $\hat{m}$:

7 $$\tau^{ \bot } \left( t \right) = \tau_{w} \left( t \right) - \left( {\tau_{w} \left( t \right) \cdot \hat{m}} \right)\hat{m}.$$

As such, the transWSS value is defined as the average over time of the absolute value of this perpendicular component:

8 $${\text{transWSS = }}\frac{1}{T}\int_{0}^{T} {\left\| {\tau^{ \bot } \left( t \right)} \right\|} \;dt.$$

OSI is evaluated to quantify temporal directional reversals of the WSS vector and to characterise regions exposed to disturbed or oscillatory near-wall flow. It is computed as follows (Moghadasi et al. 2026):

9 $${\mathrm{OSI}} = \frac{1}{2}\left( {1 - \frac{{\left\| {\int_{0}^{T} {\tau_{w} \left( t \right)dt} } \right\|}}{{\int_{0}^{T} {\left\| {\tau_{w} \left( t \right)} \right\|dt} }}} \right).$$

RRT was evaluated to characterise regions of prolonged near-wall particle residence arising from the combined effects of low time-averaged shear and oscillatory flow, computed as follows (Moghadasi et al. 2026):

10 $${\mathrm{RRT}} = \frac{1}{{\left( {{1 \mathord{\left/ {\vphantom {1 T}} \right. \kern-0pt} T}} \right)\left\| {\int_{0}^{T} {\tau_{w} \left( t \right)\;dt} } \right\|}}.$$

### Artery material properties

The carotid arterial wall is modelled as a nearly incompressible orthotropic hyperelastic soft tissue in arterial biomechanics, to capture its strongly nonlinear, direction-dependent mechanical response under physiological loading (Amabili 2018; Amabili et al. 2019; Holzapfel and Ogden 2010; Holzapfel and Weizsäcker 1998). This choice provides a compact and numerically robust constitutive representation that reproduces the experimentally observed discrepancy between axial and circumferential stiffness. This model is consistent with experimental investigations that were conducted previously in (Amabili et al. 2021; Breslavsky et al. 2020; Humphrey 2013). The wall strain-energy density *W* is formulated employing the standard decomposition into isochoric contributions describing distortional deformation and a separate volumetric term governing changes in volume and further separated into an isotropic matrix part and anisotropic fibre family parts:

11 $$W\left( {{\mathbf{C}},\,{\mathbf{a}}_{0} ,\,{\mathbf{b}}_{0} ,\,J} \right) = W_{{iso}} \left( {\bar{I}_{1} ,\,\bar{I}_{2} } \right) + W_{{fib}} \left( {\bar{I}_{4} ,\,\bar{I}_{6} } \right) + W_{{vol}} \left( J \right).$$

**Fig. 3:**
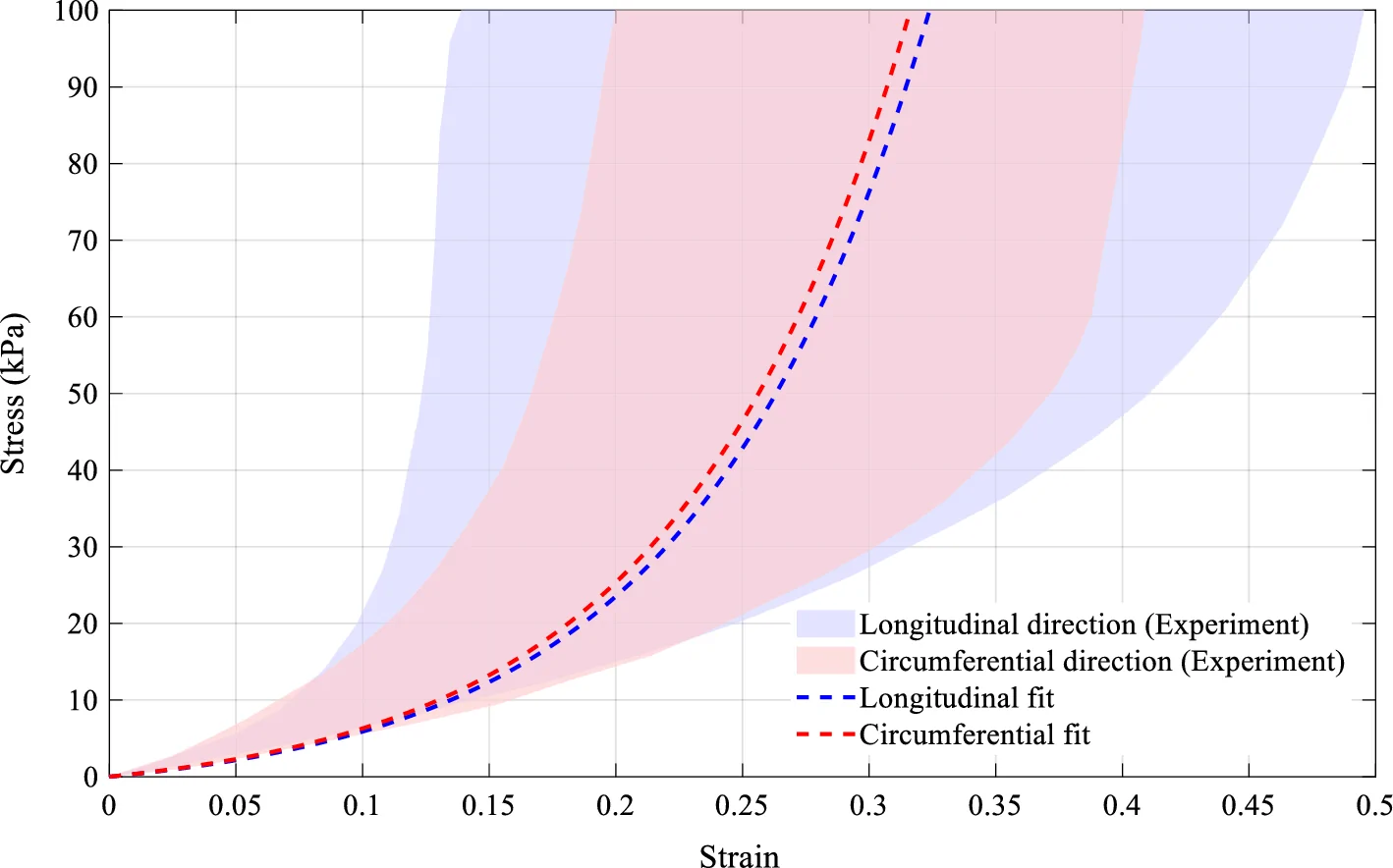
Literature reported experimental data for stress–strain relationships for both the longitudinal and circumferential directions of the common carotid artery (CCA) layer (Kamenskiy et al. 2014) compared to the fitted orthotropic hyperelastic artery

Accordingly, $W_{{iso}} \left( {\bar{I}_{1} ,\,\bar{I}_{2} } \right)$ captures the nonlinear response of the isotropic matrix (e.g. elastin-dominated behaviour at low strain), $W_{{fib}} \left( {\bar{I}_{4} ,\,\bar{I}_{6} } \right)$ captures the exponential stiffening associated with collagen recruitment along the two fibre families, and *W*_*vol*_(*J*) enforces near-incompressibility, which improves numerical conditioning and reduces volumetric locking in standard displacement-based finite element formulations. The individual contributions in Eq. 1 were implemented in ANSYS as follows (ANSYS Fluent 2023 R1 2023):

12 $$\begin{aligned} W & = \sum\limits_{i = 1}^{3} {a_{i} \left( {\overline{I}_{1} - 3} \right)^{i} } + \sum\limits_{j = 1}^{3} {b_{j} \left( {\overline{I}_{2} - 3} \right)^{j} + \frac{{c_{1} }}{{2c_{2} }}} \left[ {\exp \left( {c_{2} \left( {\overline{I}_{4} - 1} \right)^{2} } \right) - 1} \right] \\ & \quad + \frac{{e_{1} }}{{2e_{2} }}\left[ {\exp \left( {e_{2} \left( {\overline{I}_{6} - 1} \right)^{2} } \right) - 1} \right] + \frac{1}{d}\left( {J - 1} \right)^{2} , \\ \end{aligned}$$

where **C** = **F**^*T*^**F** is the right Cauchy–Green tensor, with **F** the deformation gradient and *J* = det(**F**) is the Jacobian of deformation. The isochoric tensor is defined by ${\overline{\mathbf{C}}} = J^{{{{ - 2} \mathord{\left/ {\vphantom {{ - 2} 3}} \right. \kern-0pt} 3}}} {\mathbf{C}}$, ensuring that $\overline{I}_{k}$ are volume-preserving invariants. *a*_i_, *b*_i_, *c*_1_, *c*_2_, *e*_1_, *e*_2_ are the fitted parameters derived by fitting against the data presented for the carotid artery in (Kamenskiy et al. 2014) (shown in Fig.3 ), *d* is the volumetric penalty parameter and is all shown in Table 1. The isochoric isotropic invariants follow as (Federico 2010):

13 $$\overline{I}_{1} = tr({\overline{\mathbf{C}}}),\quad \overline{I}_{2} = \frac{1}{2}\left[ {\left( {tr{\overline{\mathbf{C}}}} \right)^{2} - tr\left( {{\overline{\mathrm{C}}}^{2} } \right)} \right],$$

**Table 1:** Material property coefficients for the orthotropic hyperelastic material model (artery wall)

*a*_1_ (MPa)	*a*_2_ (MPa)	*a*_3_ (MPa)	*b*_1_ (MPa)	*b*_2_ (MPa)	*b*_3_ (MPa)	*c*_1_ (MPa)	*c*_2_ (MPa)	*e*_1_ (MPa)	*e*_2_ (MPa)	*d* (MPa^−1^)
− 3.19	13.68	64.55	1.32	− 4.61	− 19.90	14.42	1.00	12.21	1.29	2.00

These invariants describe the distortional response of the ground matrix independent of volume change. Anisotropy is introduced via two preferred structural directions **a**_0_ and **b**_0_ (unit vectors in the reference configuration), representing two fibre families aligned with the principal material axes. Their corresponding structural invariants were defined using $\overline{{\mathrm{C}}}$ as:

14 $$\bar{I}_{4} = {\mathbf{a}}_{0} \cdot \overline{{\mathbf{C}}} \,{\mathbf{a}}_{0} = \overline{{\mathbf{C}}} :\left( {{\mathbf{a}}_{0} \otimes {\mathbf{a}}_{0} } \right),\quad \bar{I}_{6} = {\mathbf{b}}_{0} \cdot \overline{{\mathbf{C}}} \,{\mathbf{b}}_{0} = \overline{{\mathbf{C}}} :\left( {{\mathbf{b}}_{0} \otimes {\mathbf{b}}_{0} } \right), ,$$

where $\overline{I}_{4}$ and $\overline{I}_{6}$ are pseudo-invariants which are introducing orthotropy by tracking stretches along two preferred directions. This invariant-based fibre representation underpins the widely used arterial constitutive frameworks introduced by Holzapfel and co-workers (Gasser et al. 2006; Holzapfel et al. 2002). For carotid modelling, the two preferred directions were chosen to coincide with the axial (longitudinal) and circumferential directions of the vessel (Holzapfel and Ogden 2010; Schiavone and Zhao 2016):

15 $${\mathrm{a}}_{0} = \left( {1,0,0} \right),\;{\mathrm{b}}_{0} = \left( {0,1,0} \right),$$

with the remaining direction implicitly treated as the thickness direction through the 3D constitutive response and the near-incompressibility constraint. Modelling the carotid wall as orthotropic is generally accurate for passive mechanics because arterial tissue exhibits pronounced direction-dependent behaviour in the circumferential and axial directions, consistent with its microstructural architecture. The effect of the activation of the vascular smooth muscle is neglected. In case of interest for this contribution for large arteries, see (Franchini et al. 2022).

Figure [3] was received; however, no citation was provided in the manuscript. Please check and confirm if the inserted citation of Fig [3] is correct. If not, suggest an alternative citation. Please note that figures should be cited in ascending numerical order in the main body of the text.I have added its right position in the text. Thank you.

### Boundary conditions

In the computational fluid domain, the inlet boundary is located at the common carotid artery, and it is subjected to a pulsatile Womersley profile (Womersley 1955). It represents a robust framework for accurate approximation of fully developed flow behaviour in the carotid artery distal to the aortic arch (Gates et al. 2018). A pulsatile Womersley velocity profile was prescribed at the inlet to represent the unsteady nature of carotid arterial flow. In large arteries, the velocity distribution is not strictly steady or fully parabolic; rather, it is governed by the balance between transient inertial and viscous forces, commonly characterised by the Womersley number. In the recent review paper (Moghadasi et al. 2026), it was shown that carotid arteries typically operate within a mid-range Womersley regime, approximately α ≈ 4 to 8, where pulsatility produces phase-dependent velocity profiles and directly influences shear-related haemodynamic markers. The Womersley formulation therefore provides a physiologically more appropriate inlet condition than a spatially uniform flat profile, particularly when WSS, OSI, and RRT are primary outputs of interest. The choice of a Womersley inlet profile is further supported by previous computational studies showing that simplified inlet assumptions can affect near-wall haemodynamic indices. Campbell et al. (2012) compared blunt, parabolic, Womersley, and measured inlet profiles in patient-specific carotid bifurcation simulations and showed that idealised velocity profiles influence WSS and OSI calculations in the carotid bulb, although patient-specific geometry and flow waveform had even larger effects. Xu et al. (2018) also assessed inlet boundary conditions in human carotid artery CFD models and reported that differences between DSA-derived and Womersley-based inlet conditions became more pronounced with increasing stenosis severity, affecting WSS, pressure, pressure gradient, and velocity predictions. A flat inlet profile imposes a uniform velocity distribution at the inlet and neglects the phase lag and radial velocity variation associated with pulsatile arterial flow. Recent sensitivity analyses have shown that differences between parabolic and Womersley profiles may become negligible after approximately two vessel diameters, but they remain relevant close to the inlet, where near-wall velocity gradients and WSS-based indices are sensitive to the imposed profile (McCarthy et al. 2025). Therefore, the Womersley profile was employed due to focusing on local WSS-based biomarkers in a pulsatile carotid flow environment, where inlet-induced differences may influence near-wall haemodynamic predictions.

To ensure physiological relevance, each outlet (internal carotid artery, ICA, and external carotid artery, ECA) was coupled to a three-element Windkessel (Resistance Compliance Resistance, RCR) model to represent the downstream vascular load beyond the finite computational domain and to mitigate non-physiological wave reflections. In general, the Windkessel effect describes how arterial compliance temporarily stores blood during systole and releases it during diastole, converting pulsatile cardiac output into a more continuous flow. This behaviour stems from the combined effects of vessel wall elasticity and downstream vascular resistance, governing the pressure–flow relationship (Westerhof et al. 2009). Figure 2c shows the applied boundary conditions in inlet and outlets.

For each outlet *i*, proximal resistance *R*_1,*i*_, distal resistance *R*_2,*i*_, and compliance *C*_*i*_ were defined such that the outlet pressure–flow relation satisfied the standard RCR governing equation. In the absence of patient-specific distal pressure measurements for the FMD cohorts, Windkessel parameters were initialised from physiological targets using a mean-pressure constraints $R_{tot,i} = {{\left( {\overline{P} - P_{ref} } \right)} \mathord{\left/ {\vphantom {{\left( {\overline{P} - P_{ref} } \right)} {\overline{Q}_{i} }}} \right. \kern-0pt} {\overline{Q}_{i} }}$, where first *P* was taken as the target mean arterial pressure and *Q*_*i*_ was prescribed according to a physiologically motivated flow split (ICA:ECA = 70:30) (Stergiopulos et al. 1998; Zarins et al. 1983). The total resistance was partitioned as $R_{1,i} = \alpha \cdot R_{tot,i}$ and $R_{2,i} = \left( {1 - \alpha } \right)R_{tot,i}$ with *α* = 0.05–0.1 (Fevola et al. 2021; Laskey et al. 1990), and *C*_*i*_ was selected via the Windkessel time constant $\tau_{i} = R_{2,i} C_{i}$ to achieve realistic pressure pulsatility. For the ICA, the proximal resistance, distal resistance, and compliance were set to *R*_1_ = 6.02 × 10^7^ Pa.s/m^3^, *R*_2_ = 1.14 × 10^9^ Pa.s/m^3^, and *C* = 8.74 × 10^–10^ m^3^/Pa, respectively. For the ECA, the corresponding parameters were *R*_1_ = 1.4 × 10^8^ Pa.s/m^3^, *R*_2_ = 2.67 × 10^9^ Pa.s/m^3^, and *C* = 3.75 × 10^–10^ m^3^/Pa. The same Windkessel parameters were applied to both the healthy and the FMD geometries. This approach ensures that the downstream vascular load remains identical across all simulations, thereby isolating the effects of FMD-associated geometric alterations on the haemodynamic response. Consequently, any observed differences in haemodynamic quantities can be attributed to changes in vascular morphology rather than variations in outlet boundary conditions. For more information related to Windkessel, interested readers are referred to the paper (Moghadasi et al. 2026).

**Fig. 4:**
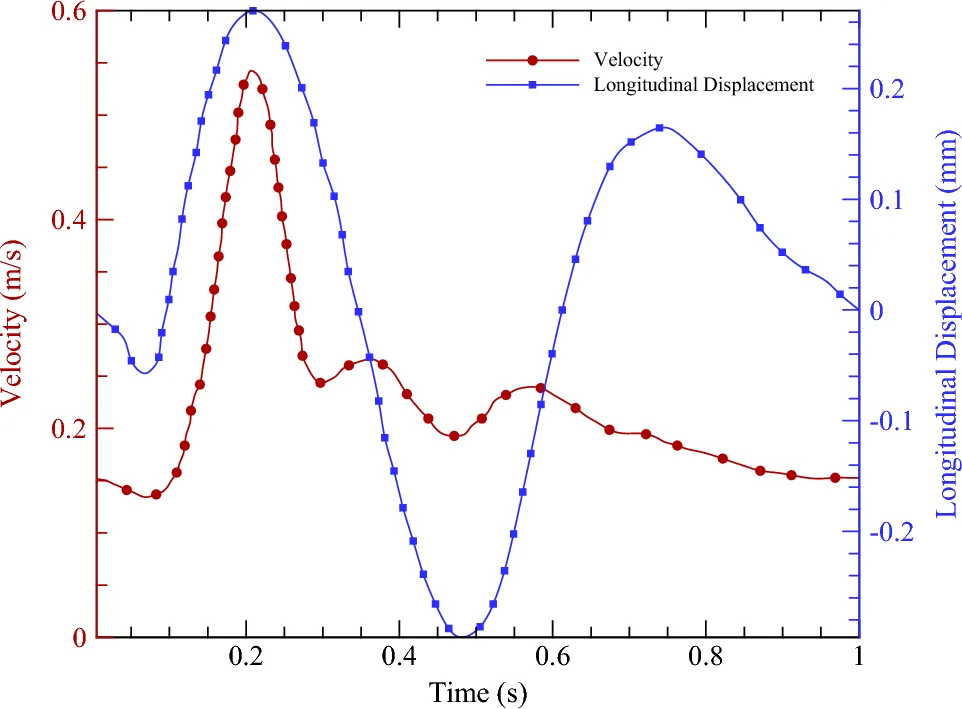
Inlet velocity and longitudinal displacement of the carotid artery models

Within this framework, the carotid artery’s physiological longitudinal (axial) wall displacement is explicitly incorporated. In vivo studies have shown that carotid arteries undergo axial motion throughout the cardiac cycle, comprising an initial systolic forward translation, a subsequent retrograde phase near peak systole, followed by a gradual anterograde recovery during diastole (Au et al. 2019). This longitudinal kinematics enables a more physically realistic representation of how arterial motion occurs when subjected to pulsatile loading, because axial displacement alters the instantaneous lumen geometry and relative near-wall flow velocities (Cinthio et al. 2006). The inlet velocity and longitudinal displacement of the artery model are presented in Fig. 4. Due to negligible interaction between the arterial wall and the surrounding tissue, the external surface of the arterial wall was modelled with a zero normal stress boundary condition (Mower et al. 1997).

### Mesh properties

In this study, the adopted computational framework for each carotid case was discretised into two coupled subdomains: a structural domain that represents the deformable arterial wall and a fluid domain that represents the intraluminal blood flow. To obtain time-periodic stability of the solutions and to minimise the impact of initial transient behaviour, each FSI model was run over three successive cardiac cycles. The quantitative analyses were performed using data extracted exclusively from the final cycle. Each 1.0 s cardiac cycle was subdivided into 200 equal time steps (∆*t* = 5 ms). At each time step, the two-way FSI coupling was iterated until convergence was achieved. Convergence was assessed based on both the reduction of fluid solver residuals (continuity, momentum, and turbulence quantities reduced below 10^–6^) and the consistency of interface variables exchanged between the fluid and structural solvers. A maximum of 30 coupling iterations per time step was imposed; however, convergence was typically achieved within this limit. Both fluid and structural domains were discretised using tetrahedral meshes to enable geometric and kinematic consistency across the interfaces. The mesh was locally enhanced at the bifurcation region, as well as near the inlet and outlet boundaries to improve the solution’s accuracy. A series of mesh-independence tests was performed for each case to confirm that the solution was independent of the mesh resolution. Table 2 describes the final mesh selections, representing a balance between solution accuracy and computational cost. Mesh convergence was evaluated by calculating the percentage variation in the area-weighted average WSS between successive mesh refinements, thereby assessing the sensitivity of the near-wall haemodynamic solution to mesh resolution. Due to the high degree of structural nonlinearity, the structural equation was solved using a sparse direct solver, which allowed for a maximum of 250 coupling iterations per step, whereas the fluid equation was discretised using second-order formulation in both space and time.

**Table 2:** Mesh convergence

Fluid part			
Mesh quality	Mesh size	Number of elements	Wall shear stress variation %
*FMD-F*			
Coarse	0.28	422,348	6.62
Fine	0.17	862,219	0.722
Extra fine	0.14	1,104,663	–
*FMD-NF*			
Coarse	0.28	432,956	7.15
Fine	0.17	900,629	0.826
Extra fine	0.14	1,200,317	–
*FMD-SNF*			
Coarse	0.28	429,571	6.89
Fine	0.17	898,762	0.808
Extra fine	0.14	1,187,482	–
Extra fine	0.20	881,351	–

Structure part			
Mesh quality	Mesh size	Number of elements	Principal stress variation %
*FMD-F*			
Coarse	0.40	206,379	5.62
Fine	0.30	532,182	0.492
Extra fine	0.20	787,935	–
*FMD-NF*			
Coarse	0.40	215,234	6.17
Fine	0.30	584,927	0.587
Extra fine	0.20	870,178	–
*FMD-SNF*			
Coarse	0.40	217,521	7.02
Fine	0.30	590,182	0.561
Extra fine	0.20	881,351	–

## Results

### Pressure

**Fig. 5:**
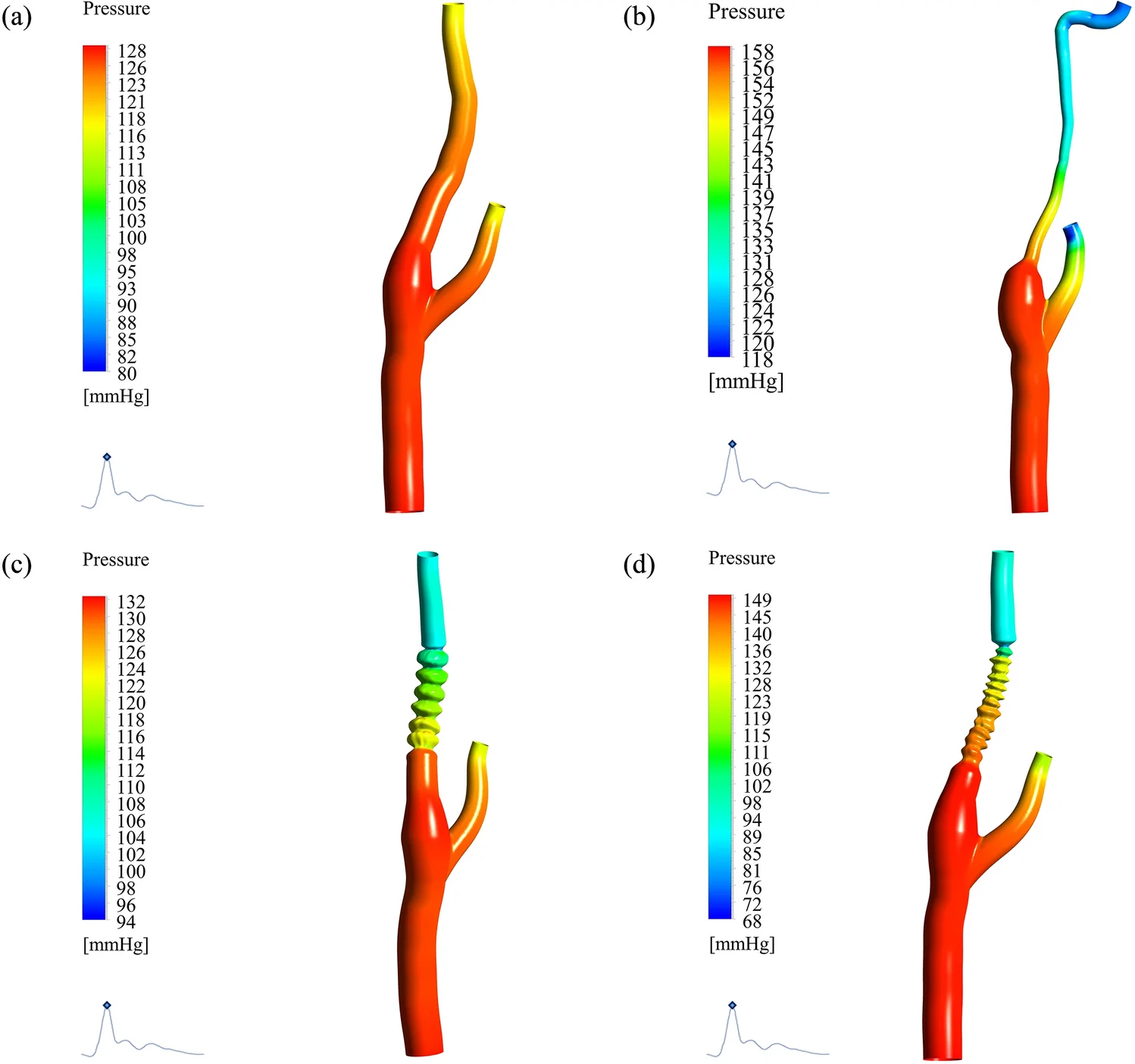
The pressure gradients at systolic phase for each model: **a** healthy carotid; **b** FMD-F model; **c** FMD-NF model; **d** FMD-SNF model

From a haemodynamic perspective, a pressure drop across a diseased carotid segment reflects energy dissipation arising from vascular resistance and disturbed flow associated with geometric irregularities. In FMD models, the characteristic series of stenotic and dilated segments introduces repeated impedance mismatches along the vessel axis, leading to cumulative axial pressure attenuation even in the absence of focal atherosclerotic narrowing (Gornik et al. 2019; Kesav et al. 2023). A reduction in distal pressure, in turn, compromises cerebral perfusion when cerebrovascular autoregulation is impaired. Previous experimental, numerical, and clinical studies of carotid geometric abnormalities have demonstrated that such alterations can induce measurable pressure drops across the affected arterial segment. It is noted that greater pressure attenuation has been implicated in an increased susceptibility to transient ischaemic symptoms in vulnerable populations (Lau et al. 2018). In this context, the progressively lower distal to proximal pressure gradients shown in Fig. 5b–d, relative to healthy geometry observed in Fig. 5a, suggest increasing haemodynamic burden and reduced efficiency of pressure transmission, indicating that more severe FMD morphology is associated with an increased risk of cerebral hypoperfusion-related events, such as TIA.

Fractional flow reserve (FFR), in coronary arteries, is an indispensable tool to identify individual coronary stenoses causing ischaemia (Ahmadi et al. 2016; Koo et al. 2011). This metric requires invasive pressure measurements under conditions of pharmacologically induced maximal hyperemia (Johnson et al. 2016; Lim et al. 2015). Direct translation of this definition to extracranial carotid artery disease is not straightforward, as standardised hyperemic protocols do not exist for the cerebral circulation (Dubs et al. 2023). Nevertheless, the underlying haemodynamic principle of FFR (a distal to proximal pressure ratio reflecting lesion-induced flow resistance) is general and not basically restricted to the coronary vasculature. Previous studies have emphasised that, in the cerebrovascular circulation, invasive translesional pressure ratios analogous to coronary artery FFR have been successfully applied to both intracranial and extracranial carotid disease. The cerebrovascular pressure ratio (CVPR) has shown strong agreement with angiography-derived quantitative flow measures and more accurately captures haemodynamically significant lesions than geometric narrowing alone (Wang et al. 2024). In a study performed by Isozaki et al. (2025), the carotid pressure ratio (CPR) correlates with cerebral vascular reserve and blood flow asymmetry, with thresholds around 0.8 identifying functionality critical lesions. These findings indicate that carotid fractional flow assessment provides a minimally invasive, functional measure of flow limitation beyond geometric narrowing alone.

**Fig. 6:**
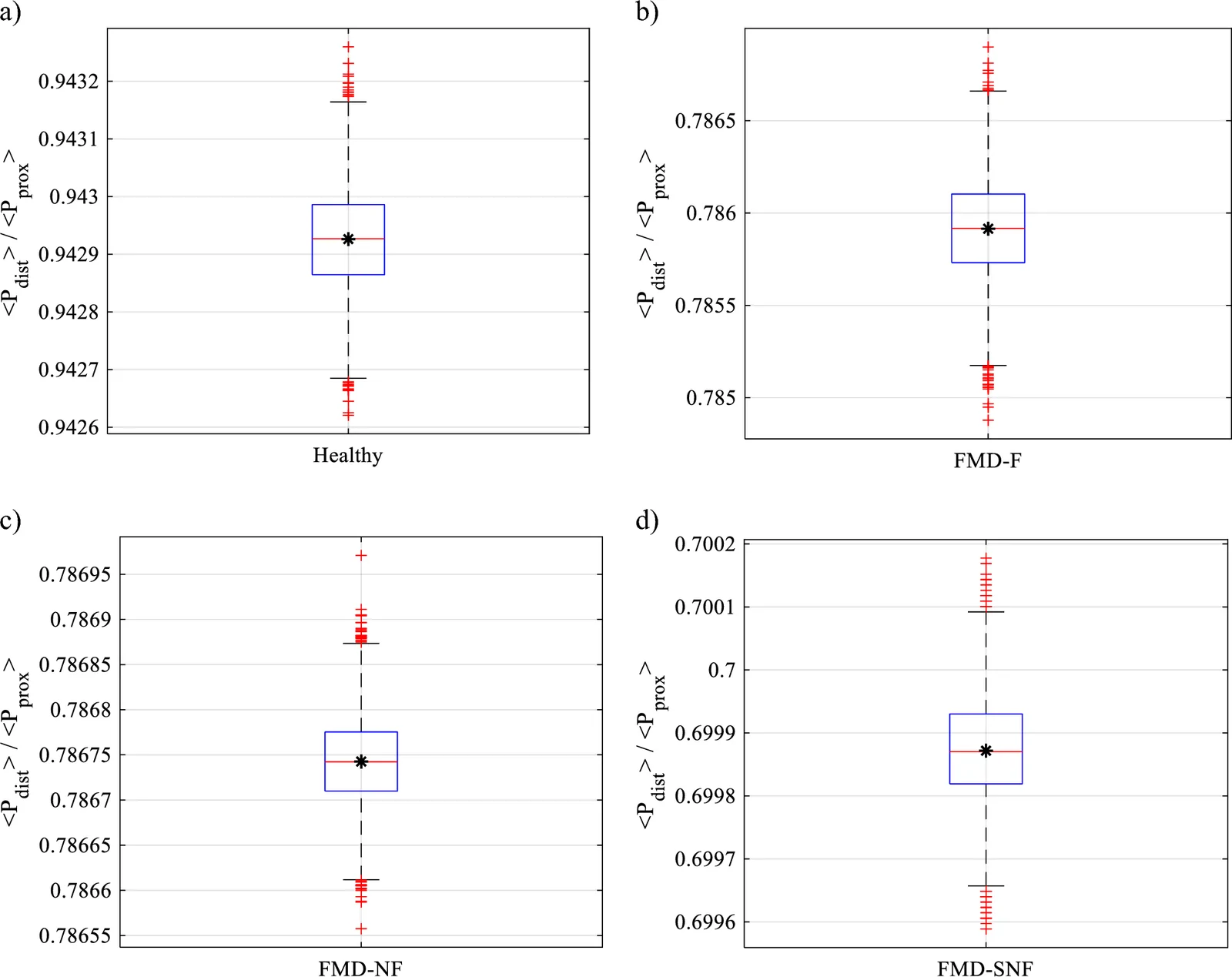
CPR values for different carotid artery models: **a** healthy; **b** FMD-F; **c** FMD-NF; **d** FMD-SNF

In this research, we therefore computed the CFD/FSI-derived distal to proximal pressure ratio (CPR), as a pressure-based functional surrogate. For each model, time-averaged pressures were calculated over multiple cross-sectional planes located proximal and distal to the FMD region, resulting in representative values of *P*_proximal_ and *P*_distal_. The ratio *P*_distal_/*P*_proximal_ thus provides a dimensionless measure of total pressure loss through the diseased segment; it quantifies the total haemodynamic impact of all individual stenotic beads together with progressive pressure loss along the FMD region. By employing multiple sampling planes rather than pointwise measurement, this approach quantifies pressure changes along the diseased segment and enables quantitative evaluation of pressure transmission between different FMD phenotypes. The use of pressure ratio based on CFD/FSI can serve as a non-invasive functional index to facilitate comparative evaluation of flow resistance across healthy and diseased carotid models. As stated previously, haemodynamic analyses of trans-stenotic pressure behaviour have indicated that a pressure ratio close to 1 represents low flow resistance and minimal haemodynamic disturbance (Isozaki et al. 2025; Nguyen et al. 2018). Consistent with this interpretation, the boxplots in Fig. 6a–d illustrate that the healthy model produced a pressure ratio close to unity, indicating minimal pressure loss along the segment. In contrast, the FMD phenotypes exhibited progressively lower CPR values. The median (interquartile range) CPR values were 0.942927 (0.942864–0.942986) for the healthy model, 0.785917 (0.785731–0.786103) for FMD-F, 0.786742 (0.786710–0.786775) for FMD-NF, and 0.699870 (0.699819–0.699930) for FMD-SNF. This trend indicates that increasing morphological severity of FMD is associated with enhanced axial pressure attenuation.

### Velocity

**Fig. 7:**
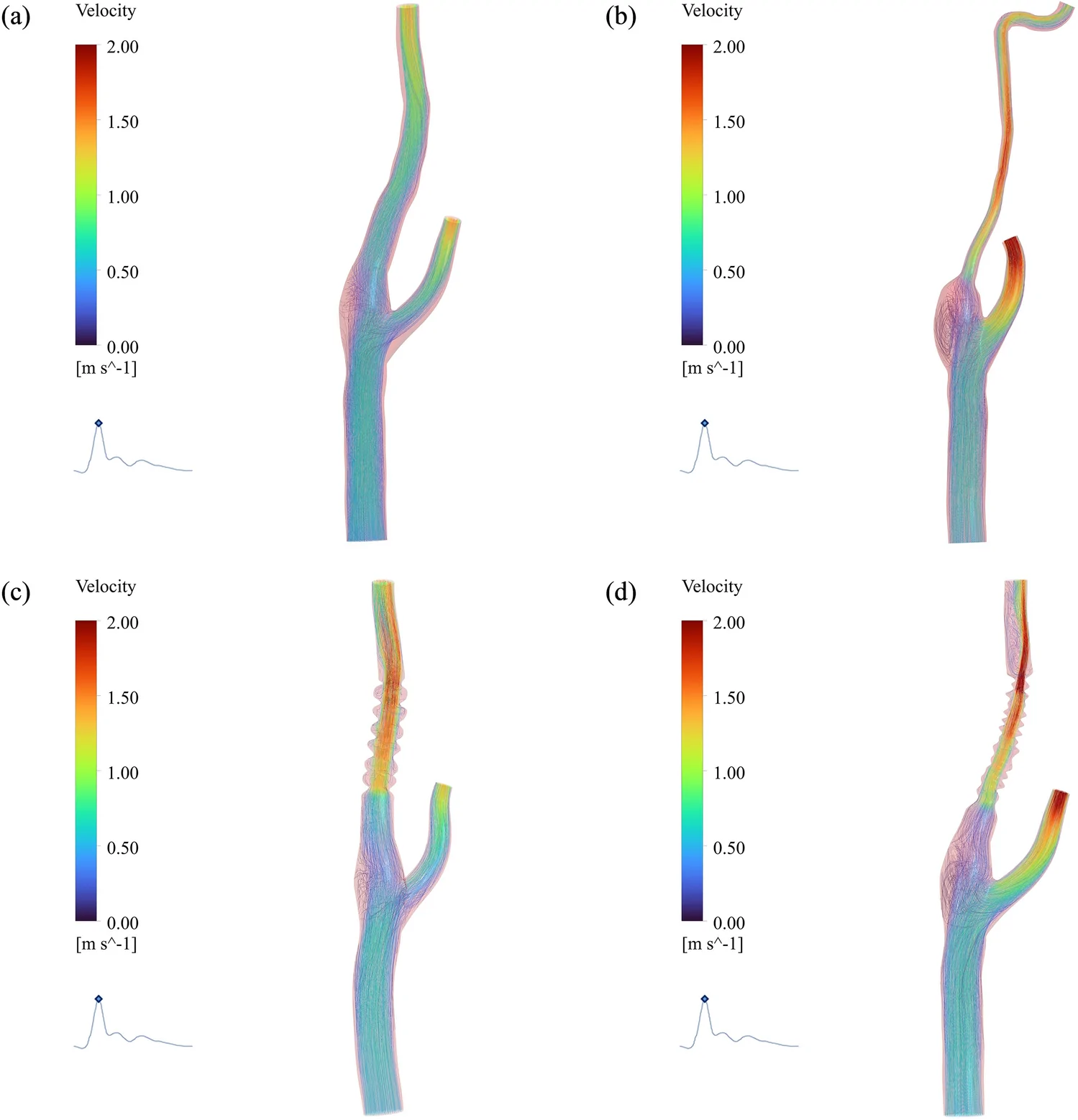
Velocity streamlines of different models at systolic phase: **a** healthy; **b** FMD-F; **c** FMD-NF; **d** FMD-SNF

The velocity streamlines serve as valuable biomarkers to diagnose FMD and evaluate disturbances of haemodynamic flow, such as the formation of post-stenotic recirculation and loss of axial flow coherence. Spatial velocity gradients induced by geometric irregularities in FMD of the carotid artery can be quantitatively measured clinically using Doppler velocities and flow turbulence downstream of stenotic segments and are often found in duplex ultrasound of carotid FMD (Chehab and Gupta 2013; Kerut et al. 2019). It is seen in Fig. 7 that, unlike the carotid artery with FMD, the velocity streamlines in the healthy carotid artery are primarily smooth and spatially coherent due to the absence of pathological luminal irregularities and preservation of normal physiological vessel geometry. As illustrated in Fig. 7a, velocity gradients develop gradually along the axial direction and maintain a uniform distribution throughout the lumen. Flow separation and slight recirculation occur at the carotid sinus due to physiological lumen dilation and associated adverse pressure gradient, with secondary flows caused by vessel curvature to affect local haemodynamics. Downstream of the bifurcation, velocity gradients dissipate quickly to allow the re-establishment of quasi-laminar flow. As a result, healthy carotids demonstrate relatively low to moderate velocity gradients compared to the significantly higher and localised velocity gradients that are commonly observed in fibromuscular dysplasia.

In the FMD-F model shown in Fig. 7b, the velocity gradient is primarily confined to the area immediately adjacent to the isolated stenotic region. Upstream of the focal lesion within the carotid sinus, recirculation zones are observed due to the adverse pressure gradients. This occurs as a result of flow separation and low-velocity vortical structures at the bifurcation. The flow then accelerates through the reduced cross-sectional area of the lumen, and a high-velocity jet is formed along with steep axial and radial gradients across the stenotic throat. Partial flow recovery occurs distal to the lesion, but some residual recirculation still exists.

On the contrary, the FMD-NF model indicates that velocity gradients distribute non-uniformly along the length of the artery because of sequential stenosis and dilatation; deceleration acceleration cycles occur frequently which generate large shear layers that are not completely dissipated between adjacent lesions (shown in Fig. 7c). The complexity of the velocity distribution therefore increases, with elongated velocity streams interacting with low velocities near the wall in adjacent regional flow disturbances observed in multifocal FMD. These velocity distributions are very similar to those measured by Doppler ultrasound, with enhanced velocities and downstream flow disturbance observed in the mid-distal internal carotid artery where limited imaging sensitivity shifts diagnostic reliance towards indirect haemodynamic markers (Ahmadpour-B et al. 2021; Cheong and Tamagnone 2025).

The FMD-SNF model is demonstrated in Fig. 7d with the most pronounced velocity gradients among all configurations. Noticeable luminal narrowing within successive stenotic segments produces intense jetting, with highly steep axial velocity gradients at stenotic throats. These jets undergo abrupt deceleration upon entering adjacent dilated regions, resulting in large-scale flow separation, recirculation zones, and sustained velocity fluctuations along the vessel wall. Such behaviour is consistent with a severe case of FMD stenoses identified clinically, where duplex ultrasound reveals elevated peak velocities and colour Doppler turbulence, reflecting indirect haemodynamic criteria of severe disease (Zhou et al. 2005). Due to severe downstream resistance, the pressure–flow balance at the bifurcation is altered, and pronounced recirculation and blood stasis occur within the carotid sinus of the FMD-SNF model.

### Transverse wall shear stress (transWSS), OSI, RRT

#### Transverse wall shear stress (transWSS)

**Fig. 8:**
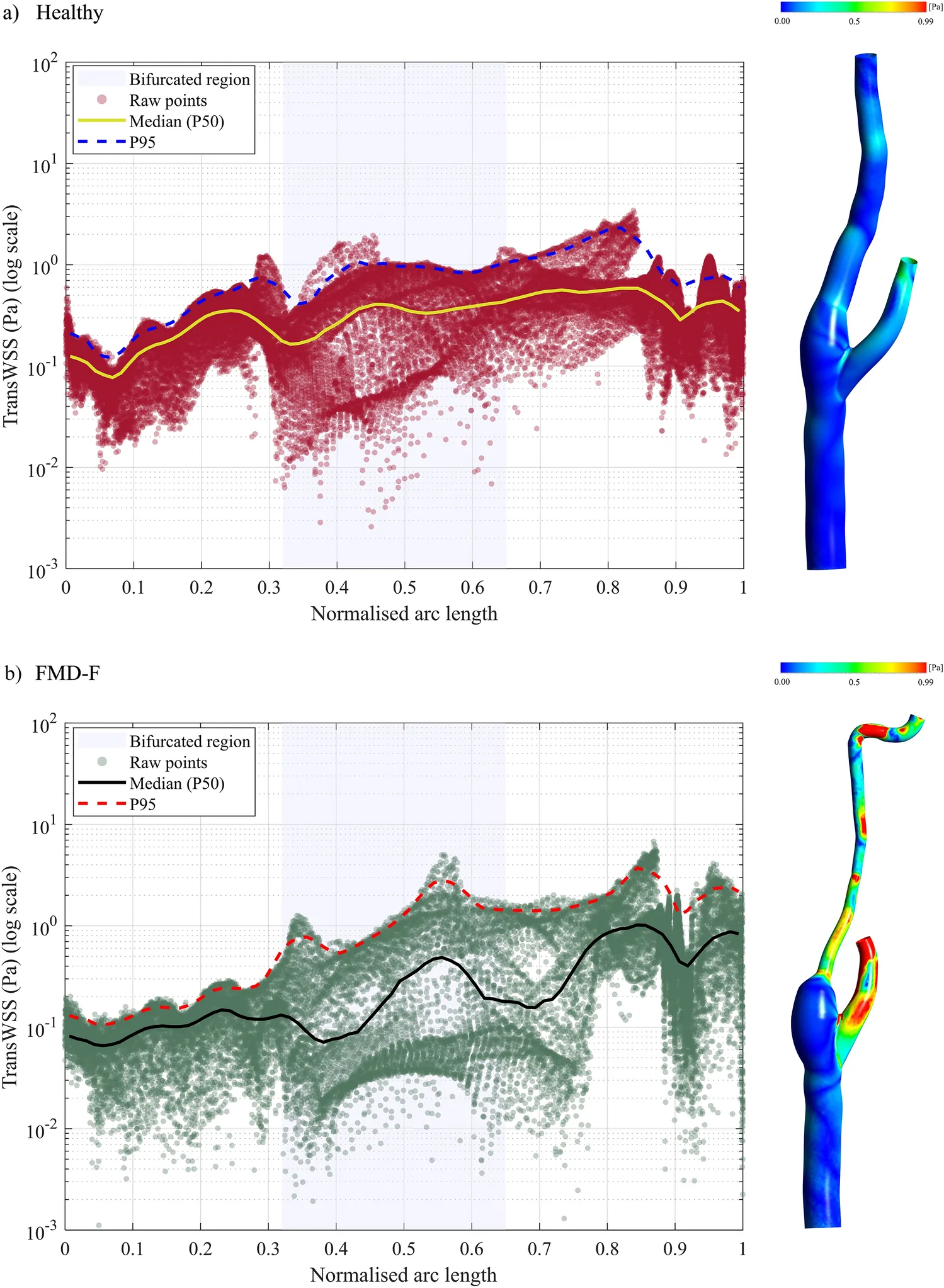

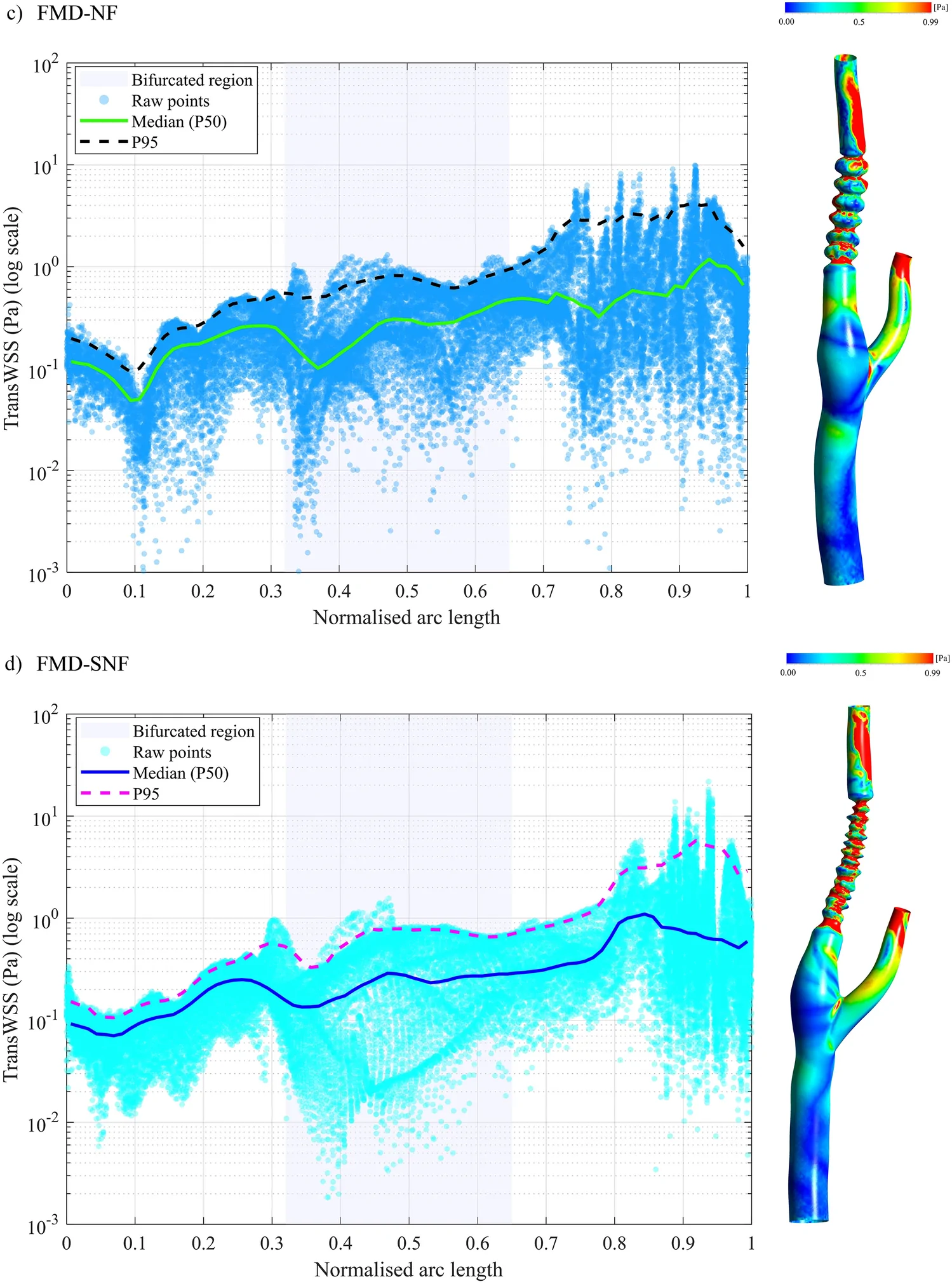
TransWSS variation and its contours along the carotid artery models: **a** healthy; **b** FMD-F; **c** FMD-NF; **d** FMD-SNF

Figure 8 depicts the spatial distribution of transWSS along the carotid artery. The transWSS contour maps for each model are also presented on the right. For each transWSS graph, the *x*-axis displays normalised arc length (*s*/*L*). Here, *s* is the cumulative distance measured along the vessel centreline, and *L* is the total centreline length of the analysed segment. Normalised arc length enables comparisons to be made directly across vessels with different lengths and levels of tortuosity. The *y*-axis represents transWSS (Pa) on a logarithmic scale; since transWSS varies over an extremely large range, this scale prevents high transWSS regions from visually masking lower magnitude yet physiologically relevant variations. The median (P50) captures the predominant transverse shear exposure along the normalised length, while the upper tail (P95) curve shows focal, extreme multidirectional shear events occurring in limited wall regions (for example, reattachment/separation regions, or secondary flow regions). The bifurcated area is shaded separately to represent the physiologically expected disturbance associated with flow division and curvature.

In the healthy model shown in Fig. 8a, transWSS remains uniformly low throughout the length of the common carotid artery, consistent with predominantly axial laminar flow. Minimal increase occurs in the area of the bifurcation, where physiological curvatures induce flow separations, localised secondary flow patterns, and near-wall directional fluctuations. TransWSS then rapidly decreases along the distal section of the artery, due to the continued alignment of the near-wall velocity field. This behaviour is also reinforced by the data obtained from the percentiles analysis; both P50 and P95 percentile lines for transWSS remain low and relatively uniform along most of the length of the artery, while a moderate increase is observed at the bifurcation. The abrupt decrease in P95 at the downstream of the artery indicates that the transient directional shear stress becomes increasingly aligned with the axial direction during each cardiac cycle. Furthermore, the narrow gap between P50 and P95 indicates a small standard deviation of the transWSS values across the arterial wall, suggesting a stable flow regime with little directional variability. This finding is in line with previous observations in (Mohamied et al. 2017).

Across FMD cases, while median (P50) transWSS values remained low along the common carotid artery, marked fluctuations were observed within the bifurcated region and its downstream. On the contrary, the upper percentile (P95) revealed pronounced disease-dependent differences, showing that FMD principally amplifies localised regions of strong multi-directional shear rather than uniformly elevating transWSS everywhere.

The FMD-F configuration, shown in Fig. 8b, is an example of how localised flow amplifies transWSS immediately distal to the focal stenosis. The median (P50) shows clearly fluctuating values post bifurcation and moderate increases in values compared to the healthy case, whereas the P95 curve shows significant peaks distal to the focal stenosis, which indicates a high degree of localised transverse shear at limited wall regions. This behaviour is consistent with a classical post-stenotic jet, in which flow acceleration through the focal stenosis triggers temporary separation from the wall and subsequent reattachment, causing transient local vortices that rapidly change the direction of wall shear (Dinh et al. 2022). Therefore, the shear disturbance is short-lived and confined to discrete wall areas rather than occurring as a continuous phenomenon.

As depicted in Fig. 8c, the FMD-NF exhibited a continuous elevation of transWSS throughout the diseased segment, in contrast to isolated areas of increased wall shear stress in the focal case. The P50 plot indicated an increase in shear stress as it progressed through the diseased segment, whereas the P95 plot remained consistently at an increased level over longer axial length. The greater separation between P50 and P95 demonstrates a bigger variation of the magnitude of transverse shear throughout localised zones, which have significantly greater multi-directional shear stresses. This type of behaviour is consistent with the creation of repeated flow acceleration, separation, and re-attachment created by the sequential “string-of-beads” geometry of the diseased segment. Overall, these patterns represent the state of disturbance of the near-wall flow, and that the secondary motions continue to be sustained, and that the flow does not fully align with the vessel axis.

Notably, the severe case (FMD-SNF shown in Fig. 8d) indicated substantially higher P50 and P95 transWSS values and a broader spatial dispersion, representing strongly disturbed, multi-directional near-wall flow, resulting in more pronounced and widespread transverse shear associated with advancing the disease to a worse situation. Importantly, transWSS captures an entirely different aspect of the haemodynamic environment than do TAWSS and Oscillatory Shear Index (OSI). OSI quantifies the reversal of shear direction over time, whereas TransWSS quantifies the directional spread of the shear vectors relative to their mean orientation. Therefore, when transWSS is elevated, it indicates that there are ongoing multi-directional shear forces present, even if there are no large-scale oscillations. The elevation of transWSS across carotid FMD phenotypes demonstrates that disease progression can be defined by an increase in shear stress, as well as the degree of near-wall flow reorientation. Multi-directional shear regimes are associated with endothelial dysfunction and pro-inflammatory and pro-thrombotic states (Rickman et al. 2023; Zhou et al. 2023a). To the best of our knowledge, the current study is the first to quantify transWSS in FMD. The findings are aligned with fundamental haemodynamic principles of flow through irregular, serially constricted vessels (Berger and Jou 2000; Bernad et al. 2012; Mohamied et al. 2017), and suggest that the string-of-bead configuration may generate secondary flow and recirculation patterns that elevate transverse shear stresses. Additionally, these effects are most pronounced in areas that may not appear critically stenotic on morphological assessment, indicating that transWSS may also be used as an early indicator of disease burden.

#### Oscillatory shear index (OSI)

**Fig. 9:**
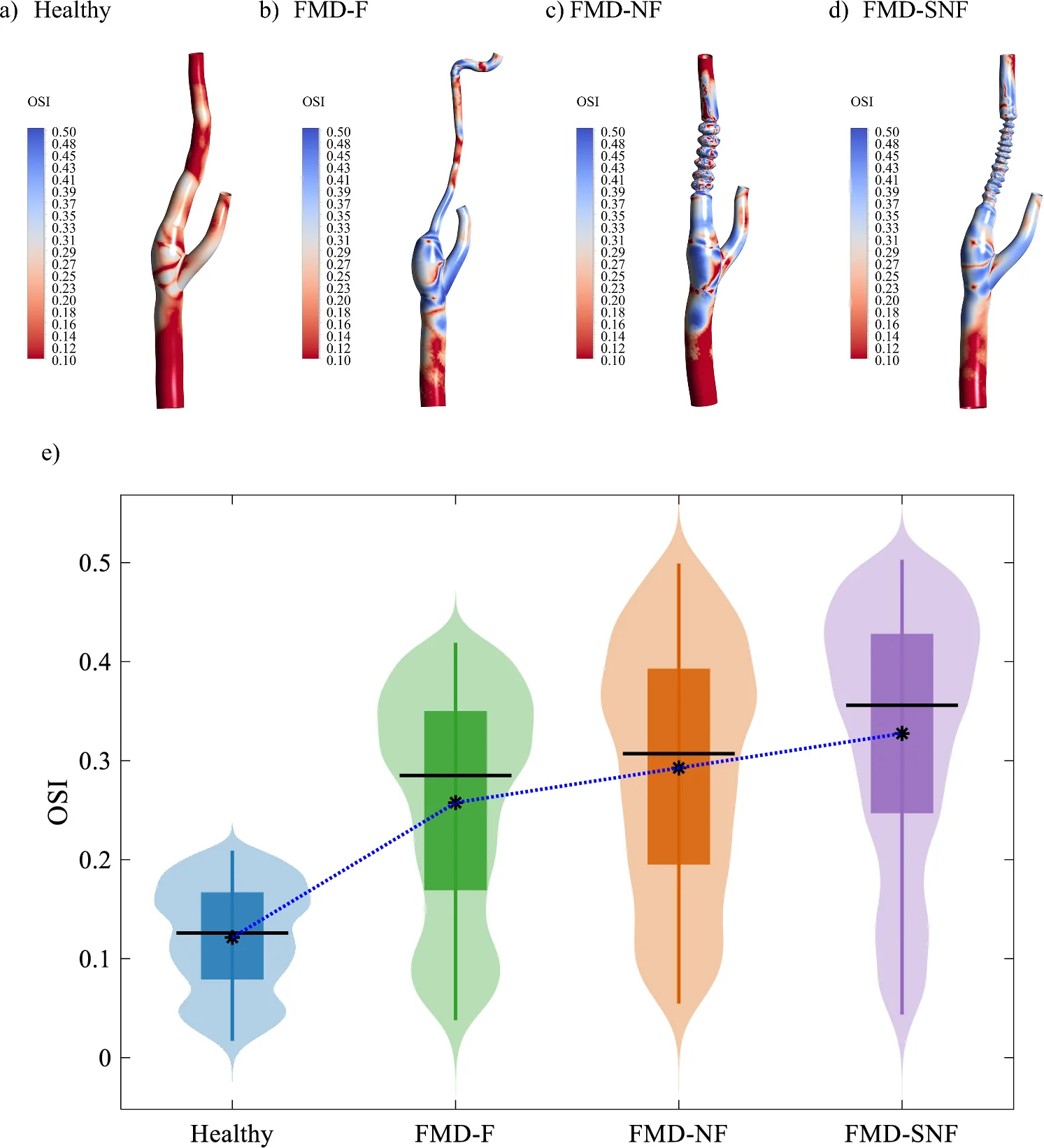
OSI contours for different models: **a** healthy; **b** FMD-F; **c** FMD-NF; **d** FMD-SNF. In the adopted colour scale, elevated OSI values are represented by blue regions, while lower OSI values are shown in red. **e** Violin plots of OSI distribution for different models

Under steady, unidirectional shear, OSI by definition is close to zero, and it increases towards 0.5 as the shear becomes bidirectional or oscillatory. Therefore, an OSI greater than 0.2 is a well-established indicator of flow separation, recirculation, and directionally unstable flow (Domanin et al. 2017; Yang et al. 2025). In the healthy carotid shown in Fig. 9a, OSI remained uniformly low throughout both the internal and external carotid arteries. The OSI values were slightly elevated near the bifurcation and at areas of curvature due to physiological flow division and secondary motion, but OSI decreased rapidly after the bifurcation, indicating stable and axially aligned shear downstream.

As illustrated in Fig. 9b, d, the FMD morphologies indicate progressively broader regions of elevated OSI at multiple locations throughout the arterial tree. In the FMD-F model (Fig. 9b), localised regions of higher OSI are observed immediately proximal to the focal stenosis, likely consistent with curvature-induced secondary flow and transient flow reversal. The disturbances occurred due to focal FMD remaining in a limited spatial area, while other parts of the artery continued to experience relatively steady shear forces. On the other hand, the FMD-NF model, depicted in Fig. 9c, is characterised by wider and longer OSI peaks at different points along the beaded segments, as a result of contraction and dilation, which produce continuous secondary flow and prevent the directional alignment of the wall shear vector. Therefore, the oscillatory shear becomes more spatially dispersed rather than being localised. The most significant disturbance is found in FMD-SNF (shown in Fig. 9d), where there are large areas of high OSI along a significant portion of the arterial length. Elevated OSI values result from the cumulative influence of consecutive geometric irregularities that have caused chronic directional oscillations of the shear field, and therefore, chronically unstable near-wall flow for a long time.

The violin plots in Fig. 9f further quantify these trends by illustrating the full distribution of OSI values for each model. Uniformly stable shear is indicated by a low median value and a relatively small range in OSI values for the healthy model. In the FMD-F phenotype, the median OSI shows a slightly upward shift with increased dispersion, reflecting the emergence of localised regions of pronounced oscillatory shear. In the FMD-NF model, the OSI values show a wider range than in the FMD-F, with higher median and interquartile range values, indicating that areas of shear reversal are more spatially extensive. The OSI values in the FMD-SNF model represent the highest median OSI value and the largest amount of variability among all four phenotypes; thus, it is apparent that as the disease progresses, the oscillatory shear becomes stronger and more pervasive. For the OSI, statistical analysis demonstrated significant differences in the spatial haemodynamic distributions among the investigated models. A Kruskal–Wallis test confirmed an overall statistically significant difference across all groups (*p* < 0.001). Subsequent pairwise Mann–Whitney U tests with Bonferroni correction further demonstrated that all pairwise comparisons remained statistically significant (corrected *p* < 0.001). These findings quantitatively support the distinct OSI distribution patterns observed in the violin plots and indicate that the different FMD phenotypes generate substantially different oscillatory shear environments along the carotid arterial wall.

#### Relative residence time (RRT)

Increased RRT indicates normally slow-moving or recirculating flow, resulting in a longer interaction time with the endothelial layer. Such an environment is generally associated with flow stagnation and higher susceptibility towards thrombosis and inflammation (Moghadasi et al. 2024a; Piek et al. 2026).

**Fig. 10:**
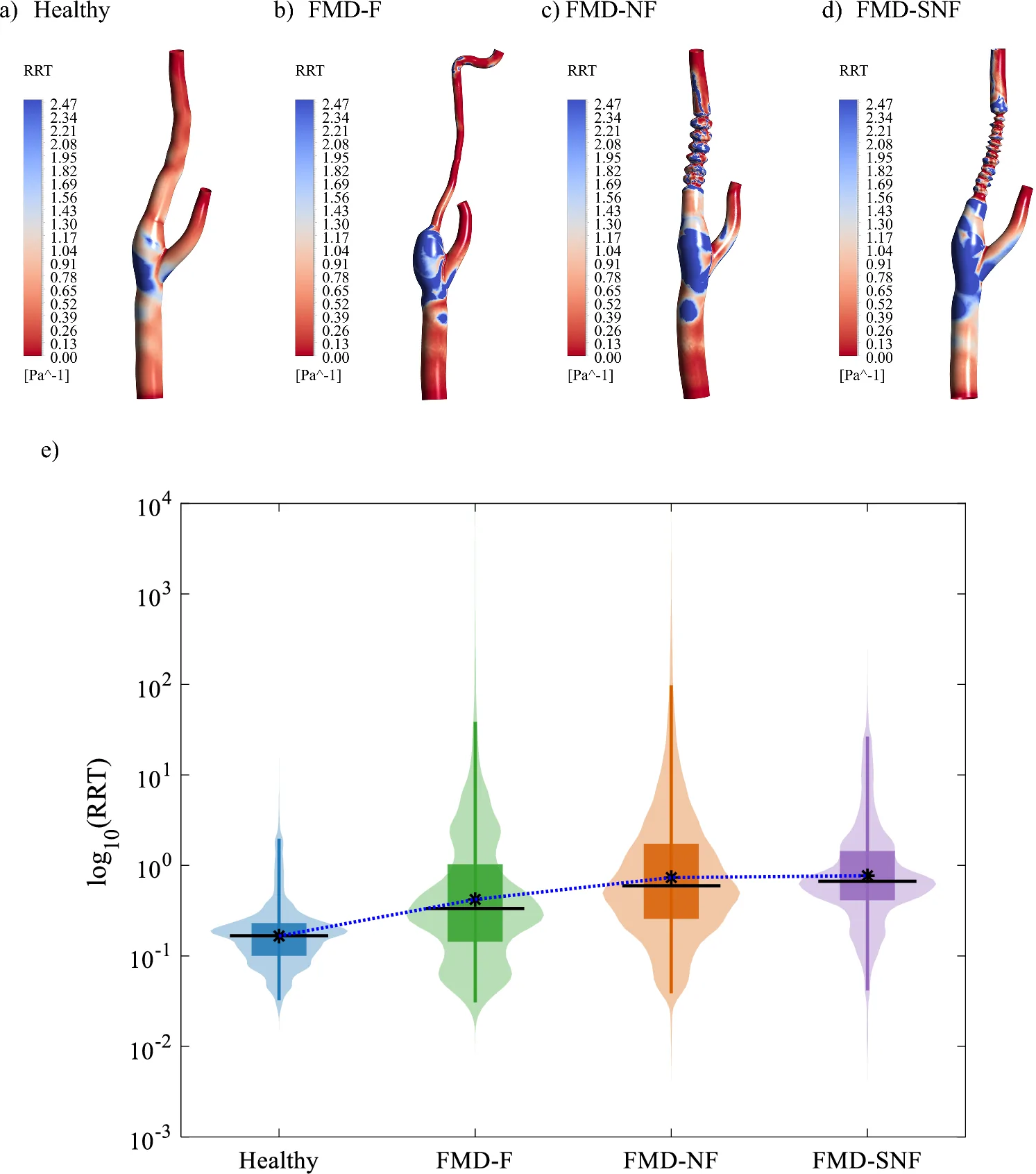
RRT contours for different models: **a** healthy; **b** FMD-F; **c** FMD-NF; **d** FMD-SNF. In the adopted colour scale, elevated RRT values are represented by blue regions, while lower RRT values are shown in red. **e** Violin plots of RRT for different models

In the healthy carotid artery (shown in Fig. 10a), the majority of the artery regions were characterised by low RRT values. RRT elevation was limited to the area of the bifurcation which would be expected to experience some degree of physiological recirculation. Downstream of the bifurcation, a rapid decrease in RRT values indicates the absence of persistent flow stagnation under predominantly unidirectional shear. The presence of fibromuscular dysplasia progressively altered these patterns. It is seen in Fig. 10b that moderate RRT elevations in the FMD-F model are observed within the carotid sinus, the region proximal to the focal narrowing section and the distal expansion regions, where local flow deceleration and curvature-induced secondary motion promote transient near-wall residence. On the contrary, elevated RRT was not observed within the stenotic segment itself, as the accelerated flow through the narrowing segment generates high wall shear stresses and rapid near-wall transport, thereby reducing residence time. In contrast, the non-focal phenotypes depicted in Fig. 10c and d (FMD-NF and FMD-SNF) indicate wider areas of elevated RRT along the beaded segment. A repetitive sequence of constriction and dilation in the internal carotid artery results in successive flow deceleration and reattachment, which creates recirculating zones that increase the near-wall time for fluid particles along the vessel length. Consequently, higher levels of RRT are found in wider areas rather than localised.

The violin plots, shown in Fig. 10f, are employed to provide a quantitative summary of the trends. Because RRT spans multiple orders of magnitude and is skewed towards elevated values, the violin plots are presented on a logarithmic scale to enable meaningful visualisation of both the central tendency and the long-tail behaviour without domination by extreme values. A clear, monotonic increase is observed for both the central tendency and dispersion of RRT as the graph progresses from healthy to the severe form of FMD. In the case of a healthy individual, there is a very small standard deviation with a low median value, which can be interpreted as minimal particle residence near the wall. For FMD-F, an upward trend with a relatively modest spread is noted, indicating that although prolonged residence time exists, it is primarily localised. FMD-NF displays a broader distribution with elevated median and interquartile ranges, indicating more extensive near-wall stagnation. Ultimately, the highest median is observed for FMD-SNF, which indicates that with increased disease severity, prolonged residence becomes stronger and spatially pervasive. Statistical analysis of RRT similarly revealed significant differences in the spatial haemodynamic distributions between the studied models. Overall group comparison using the Kruskal–Wallis test demonstrated a statistically significant difference across all groups (*p* < 0.001), while subsequent pairwise Mann–Whitney U analyses with Bonferroni correction confirmed statistical significance for all intergroup comparisons (corrected *p* < 0.001). These results substantiate the pronounced differences in near-wall flow stasis and disturbed haemodynamic transport behaviour associated with the different FMD morphologies, as reflected by the RRT violin plot distributions.

### Arterial deformation and stress

**Fig. 11:**
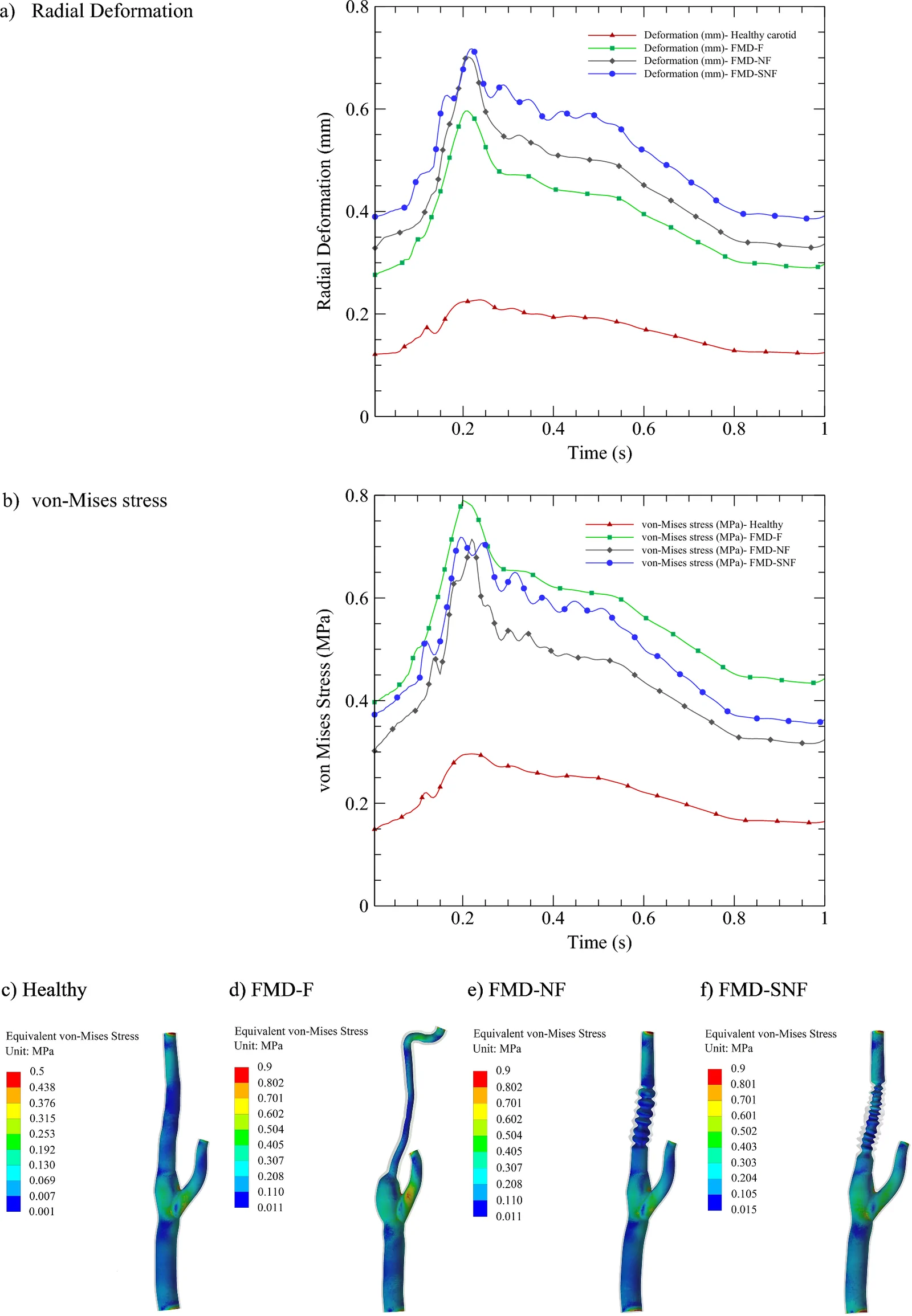
The variation of radial arterial wall deformation and von-Mises stress for different models shown in (**a**) and (**b**). Contours of von-Mises stress for: **c** healthy; **d** FMD-F; **e** FMD-NF; **f** FMD-SNF

As depicted in Fig. 11a, radial deformation increases progressively with disease severity, with FMD-SNF representing the largest systolic expansion, followed by FMD-NF and FMD-F, while the healthy carotid demonstrates substantially lower displacement throughout the cardiac cycle. Peak deformation in FMD-SNF is approximately threefold greater than in the healthy model, indicating significant alteration in wall mechanical response under pulsatile loading. Transmural pressure, defined as the difference between intraluminal and external pressure (*P*_*tm*_ = *P*_*inside*_-*P*_*outside*_), represents the effective mechanical load acting across the arterial wall. It is the primary driver of radial distension during the cardiac cycle (Dobrin 2011; Khamdaeng et al. 2012). While transmural pressure provides the principal driving load for wall distension, the FMD-SNF configuration amplified radial deformation in the upstream sinus region because the extended, irregular beaded morphology altered downstream mechanical impedance and geometric compliance differences.

In contrast, peak von-Mises stress, shown in Fig. 11b, does not follow the same monotonic trend. Interestingly, the FMD-F model shows the highest systolic wall stress, exceeding that of both non-focal configurations. With identical material properties and boundary conditions, this suggests that abrupt geometric transitions and stiffening in focal disease induce stronger local stress concentration, whereas more distributed morphological irregularities in non-focal cases promote broader deformation with comparatively lower peak stress amplification. Fig. 11c–f illustrates von-Mises stress variations for the healthy and FMD geometries. The stress remains low and spatially uniform along the healthy model, with a moderate increase around the bifurcation. However, in the FMD-F model (Fig. 11d), higher stress emerges near the carotid bifurcation, with additional stress amplification extending along the ICA and at the proximal ECA origin. This reflects the sensitivity of wall stresses to sharp geometric gradients and pressure redistribution across geometric constriction. These findings are consistent with established vascular biomechanics literature, where sharp stenotic gradients are known to elevate structural stress despite lower global displacement (Li et al. 2006; Tang et al. 2003). On the other hand, more distributed stress patterns are observed along the beaded segments of non-focal FMD models, shown in Figs. 11e and f. The reported stress magnitudes (0.7–0.8 MPa in diseased models) remain within experimentally reported upper physiological ranges for carotid arterial tissue.

## Discussion

The present findings provide a haemodynamic interpretation of the clinical behaviour of carotid FMD. FMD is often asymptomatic, but it may also be associated with headache, transient ischaemic attack, ischaemic stroke, and haemorrhagic complications. These clinical manifestations are consistent with the notion that FMD is not only an anatomical abnormality but also a functional vascular disorder capable of altering local and downstream haemodynamics (Kesav et al. 2023). In this context, the reduction in CPR observed across the FMD phenotypes suggests that the beaded morphology produces measurable axial pressure attenuation. This is clinically relevant because pressure loss across stenotic or irregular arterial segments reflects haemodynamic burden and may contribute to impaired distal perfusion, particularly in more severe or diffuse disease. Additionally, pressure gradient assessment is also recognised clinically as useful for determining the haemodynamic significance of FMD-related stenoses (Isozaki et al. 2025; Petropoulos et al. 2024). The FMD-SNF model showed the lowest CPR, indicating the greatest pressure attenuation among the studied phenotypes. On the contrary, the observed increases in disturbed-flow markers, including elevated OSI, RRT, and multi-directional shear behaviour, also have important mechanistic implications. Regions of low or oscillatory shear are associated with prolonged near-wall residence, endothelial dysfunction induced by disturbed flow, and altered vascular wall biology. Although FMD is not primarily an atherosclerotic disease, abnormal shear environments are still clinically relevant because endothelial dysfunction, local reversal flow, and prolonged residence time can promote thromboembolic susceptibility or contribute to unfavourable vascular remodelling such as tortuosity and aneurysm. This interpretation is consistent with broader carotid haemodynamic studies showing that WSS-related indices, OSI, and RRT can provide risk-relevant information beyond anatomical stenosis alone (Baeyens et al. 2016; Chen et al. 2024; Narula et al. 2018). These findings may also help explain why some patients with carotid FMD develop neurological symptoms despite the absence of conventional atherosclerotic plaque. The studies have reported that FMD is associated with neurovascular manifestations including pulsatile tinnitus, TIA, stroke, aneurysm, and dissection. In the US Registry for FMD, aneurysm and dissection had occurred in a substantial proportion of patients by registry entry, supporting the need to consider FMD as a generalised vascular disease with potential cerebrovascular consequences (Dicks et al. 2021; Kadian-Dodov et al. 2018; Olin et al. 2012).

In addition to the geometric effects discussed above, it is also relevant to interpret the present findings in the context of pulsatile flow behaviour, characterised by the Womersley number. As discussed in the recent review paper (Moghadasi et al. 2026), carotid arteries typically operate within a moderate Womersley regime (α ≈ 4 to 8), where both inertial and viscous effects influence the velocity profiles. Previous studies have shown that Womersley-based inflow conditions can affect velocity phase distribution, WSS, and recirculation patterns, particularly in stenotic arteries (Udupa et al. 2024, 2025). However, these effects primarily modulate the temporal characteristics of the flow, whereas the dominant haemodynamic trends are largely governed by vascular geometry (Campbell et al. 2012; Steinman 2002). Therefore, within the physiological carotid Womersley range, the principal trends reported here are expected to remain robust.

The present FSI results therefore complement clinical imaging by translating morphological changes into functional haemodynamic findings. Overall, these findings may help support future risk stratification frameworks for carotid FMD, particularly in patients whose symptoms or risk profile is not fully explained by luminal appearance alone.

## Conclusion

In this study, a 3D two-way FSI technique was employed to investigate the coupled haemodynamic and structural behaviour of carotid FMD. Patient-specific geometries representing healthy, focal (FMD-F), non-focal (FMD-NF), and severe non-focal (FMD-SNF) phenotypes were analysed. The arterial wall was modelled using an orthotropic hyperelastic formulation, and physiologically consistent inlet and outlet conditions were imposed via pulsatile velocity flow and a Windkessel model, respectively. The following are some key findings that have been identified as a result of the analysis:

1. In the healthy artery, flow remained mainly axisymmetric with quick distal realignment. However, flow acceleration was consistently observed within narrowed regions of FMD models due to their special geometric constriction. Velocity disturbances of FMD-F remained spatially localised, with post-narrowing fluctuations, whereas non-focal models showed secondary flow structures and non-uniformity along beaded segments. The FMD-SNF model produced the most extended regions of disturbed flow, characterised by enhanced flow instability.
2. A progressive burden on pressure transmission was imposed by FMD through the carotid artery. Repeated impedance mismatches occurred owing to the serial narrowing and dilation inherent to FMD which resulted in cumulative axial pressure attenuation. The progressively lower distal to proximal pressure ratio in FMD morphology is associated with escalating haemodynamic compromise and may contribute to the high vulnerability of cerebral hypoperfusion-related events.
3. Low and spatially uniform transWSS was observed in the healthy carotid model. OSI and RRT remained low, reflecting minimal oscillatory shear and near-wall residence. In contrast, FMD-F showed localised elevation associated with amplified transWSS immediately distal to the focal stenosis. OSI and RRT elevations were primarily observed in the sinus and regions adjacent to the narrowing segment. In FMD-NF morphology, higher transWSS was spatially distributed along the beaded region, with more persistent oscillatory shear regions and sustained residence time prolongation along the diseased segment. FMD-SNF demonstrated higher median and high-percentile transWSS, indicative of multi-directional near-wall flow. This was accompanied by a pronounced increase in OSI and RRT values.
4. FMD geometric irregularities in carotid arteries amplified wall deformation and mechanical stress concentrations. The healthy carotid indicated the lowest deformation and von-Mises stress. In FMD-F, while deformation remained moderate, von-Mises stress was the highest among all models, representing that abrupt geometric transitions may induce strong local stress concentration. On the other side, non-focal morphologies (FMD-NF and FMD-SNF) demonstrated broader deformation with comparatively lower peak von-Mises stress.

## Data Availability

No datasets were generated or analysed during the current study.
